# Targeting super elongation complex-driven RNA polymerase II elongation reduces plaque vulnerability

**DOI:** 10.1038/s41392-026-02962-4

**Published:** 2026-09-17

**Authors:** Karoline E. Kokot, Sebastian Reichardt, Christian Menedo, Stephan Erbe, Jasmin M. Kneuer, Kaylin C. A. Palm, Julian Hoba, Michael Andritschke, Mirko Katzmann, Annika Reuser, Moritz von Scheidt, Michal Mokry, Junjie Xiao, Bilal N. Sheikh, Nora Klöting, Ulrich Laufs, Jes-Niels Boeckel

**Affiliations:** 1 Klinik und Poliklinik für Kardiologie, Universitätsklinikum Leipzig, Leipzig, Germany; 2 Division Laboratories, Pharmacy, and Biomedical Genetics, Central Diagnostics Laboratory, University Medical Centre Utrecht, Utrecht, The Netherlands; 3 Department of Cardiology, TUM Klinikum Deutsches Herzzentrum, Technical University Munich, Munich, Germany; 4 Deutsches Zentrum für Herz- und Kreislaufforschung (DZHK), Partner Site Munich Heart Alliance, Munich, Germany; 5 Institute of Cardiovascular Sciences, Shanghai Engineering Research Center of Organ Repair, Joint International Research Laboratory of Biomaterials and Biotechnology in Organ Repair (Ministry of Education), School of Life Science, Shanghai University, Shanghai, China; 6 Helmholtz Institute for Metabolic, Obesity and Vascular Research (HI-MAG) of the Helmholtz Zentrum München at the University of Leipzig and University Hospital, Leipzig, Germany; 7 German Center for Cardiovascular Research (DZHK), Partner Site Heidelberg, Heidelberg, Germany

**Keywords:** Cardiology, Molecular biology

## Abstract

Atherosclerotic plaque rupture is a major cause of myocardial infarction and stroke, yet the mechanisms governing plaque stability remain incompletely understood. Endothelial activation can trigger endothelial-to-mesenchymal transition, a program linked to endothelial dysfunction and lesion vulnerability. Here we investigated whether transcriptional pause release and RNA polymerase II elongation constitute an early regulatory layer that promotes endothelial-to-mesenchymal transition and atherosclerosis. Analysis of human plaque single-cell transcriptomics indicated increased expression of super elongation complex components in endothelial cells with a transition signature. In primary human endothelial cell models, pharmacological inhibition of the super elongation complex attenuated the induction of mesenchymal markers. AFF4, pCDK9, and pSMAD2/3 showed physical interaction during endothelial transition. Genome-wide profiling of RNA polymerase II occupancy revealed reduced promoter-proximal pausing during early transition, accompanied by a rapid increase in nascent transcriptional elongation rates. Super elongation complex inhibition restored pausing and suppressed fast-responding transition-associated target genes. In a human cardiac organoid model, inhibition of the super elongation complex prevented EndMT-induced fibrillar collagen deposition and prevented the loss of beating rate. In a hyperlipidemic *Pcsk9* gain-of-function mouse model, super elongation complex inhibition administered both prophylactically and therapeutically after established atherosclerosis reduced plaque burden and reduced features of plaque vulnerability. Finally, analysis of 1048 human plaque segments from the Athero-Express biobank showed significant associations between the elongation axis and multiple vulnerability-related plaque traits. Together, these findings identify rapid transcriptional elongation as a mechanistic driver of endothelial plasticity and features of plaque vulnerability and support targeting the elongation machinery as a potential strategy to reduce features of plaque vulnerability in atherosclerotic disease.

## Introduction

Atherosclerosis is a chronic arterial disease and a leading cause of morbidity and mortality worldwide.^1^ The arterial endothelium forms the first protective barrier against atherogenic stressors, but under sustained insult it can become dysregulated. Endothelial dysfunction is an early driver of plaque development, and endothelial cells (ECs) can even undergo phenotypic change into mesenchymal-like cells.^2–6^ This process, known as endothelial-to-mesenchymal transition (EndMT), has been shown to contribute to atherosclerosis by promoting EC dysfunction and plaque progression.^7–9^ In fact, cardiovascular risk factors and plaque microenvironmental stress (e.g., chronic inflammation, disturbed flow, and hypoxia) are known to trigger EndMT in ECs.

Once initiated, EndMT leads to profound cellular changes that can exacerbate plaque development. EndMT-derived mesenchymal cells (often fibroblast-like) secrete extracellular matrix components and pro-inflammatory factors influencing plaque architecture and stability.^7,10^ Lineage-tracing and histopathology studies have confirmed that “transitioning” cells co-expressing endothelial and mesenchymal markers are present in atherosclerotic lesions. Notably, the extent of EndMT in plaques correlates with lesion complexity and instability.^7^ In advanced atherosclerotic plaques, a higher abundance of EndMT-positive cells is associated with fragile, unstable plaques prone to rupture.^7^ These findings underscore EndMT as a pathologic program that not only contributes to plaque growth but also potentially precipitates adverse clinical events.^8,11^

Despite the growing recognition of EndMT in atherogenesis, the upstream mechanisms that link vascular risk factors to this endothelial phenotypic shift remain incompletely understood. Prior studies have largely focused on classical signaling pathways (such as TGF-β/SMAD, Notch, and BMP signaling) as drivers of EndMT.^9,12–16^ While these pathways activate transcription factors and epigenetic changes that initiate EndMT,^6,17^ much less is known about how the transcriptional machinery itself is mobilized during EndMT. Converting an EC to a mesenchymal cell requires coordinated upregulation of a broad gene program, suggesting that fundamental gene regulatory mechanisms are at play. In particular, the control of transcription at the RNAPII elongation stage has emerged as a crucial, albeit underappreciated, layer of gene regulation in dynamic cellular transitions.^18^

Cells often prime critical genes for rapid activation by loading RNAPII at promoters in a paused state, allowing for prompt gene expression upon stimulation.^19^ This promoter-proximal pausing and subsequent release of RNAPII is a well-established mechanism to tightly control the timing and amplitude of transcriptional responses.^18,20^ Many stress-responsive and developmental genes are regulated at this pause-release checkpoint.^19,21^ For example, in resting immune cells, RNAPII initiates transcription of pro-inflammatory genes but stalls near the promoter, only resuming elongation when an inflammatory stimulus occurs.^22,23^ The transition from paused to actively elongating RNAPII is governed by the positive transcription elongation factor b (P-TEFb), a CDK9/cyclin T kinase complex that phosphorylates RNAPII and pause-inducing factors to enable productive elongation.^20^ Notably, P-TEFb does not act alone; it is recruited to target genes as part of large co-factor assemblies that integrate signaling cues with the transcriptional apparatus.^20,21^

One of the key regulators of P-TEFb–mediated pause release is the super elongation complex (SEC). The SEC is a multiprotein complex assembled on scaffold proteins *AFF1* and *AFF4* (members of the AF4/FMR2 family), which tether P-TEFb to transcription sites and recruit additional elongation factors (such as *ENL, AF9*, and *ELL* proteins).^20,24,25^ Through this mechanism, the SEC serves as a master regulator of rapid transcriptional induction. Indeed, the AFF1/AFF4-containing SEC exhibits robust RNAPII C-terminal domain kinase activity and is uniquely required for the prompt activation of certain stress-responsive genes (for instance, only SEC is required for proper induction of the HSP70 heat shock gene upon stress).^20^ Genome-wide studies have further shown that AFF4/SEC plays a dominant role in facilitating immediate early gene expression in mammalian cells.^26^ The importance of SEC-mediated transcription elongation has been demonstrated in developmental biology and cancer pathogenesis, and CDK9 in particular is a target of current drug development, examples being BAY1143572/Atuveciclib (Bayer), PHA-767491 (Pfizer), LY2857785 (Eli Lilly) or AZ5576 (AstraZeneca),^27^ but its role in vascular disease has not been explored. To date, no studies have addressed whether this transcription elongation machinery contributes to endothelial dysfunction or atherogenesis.

We hypothesized that atherosclerosis-relevant stress cues initiate endothelial-to-mesenchymal transition through a rapid shift in transcriptional kinetics, specifically RNA polymerase II pause release and processive elongation driven by the super elongation complex. To test this, we integrated human plaque transcriptomics, primary human endothelial cell transition models, genome-wide measurements of polymerase pausing and nascent transcription, and an in vivo hyperlipidemic atherosclerosis model. This multi-layered strategy allowed us to connect transcriptional elongation control to endothelial plasticity and plaque vulnerability across experimental and human disease contexts.

## Results

### The components of the super elongation complex are increased by EndMT and in EndMT-positive ECs of human atherosclerotic plaque sections

**Fig. 1:**
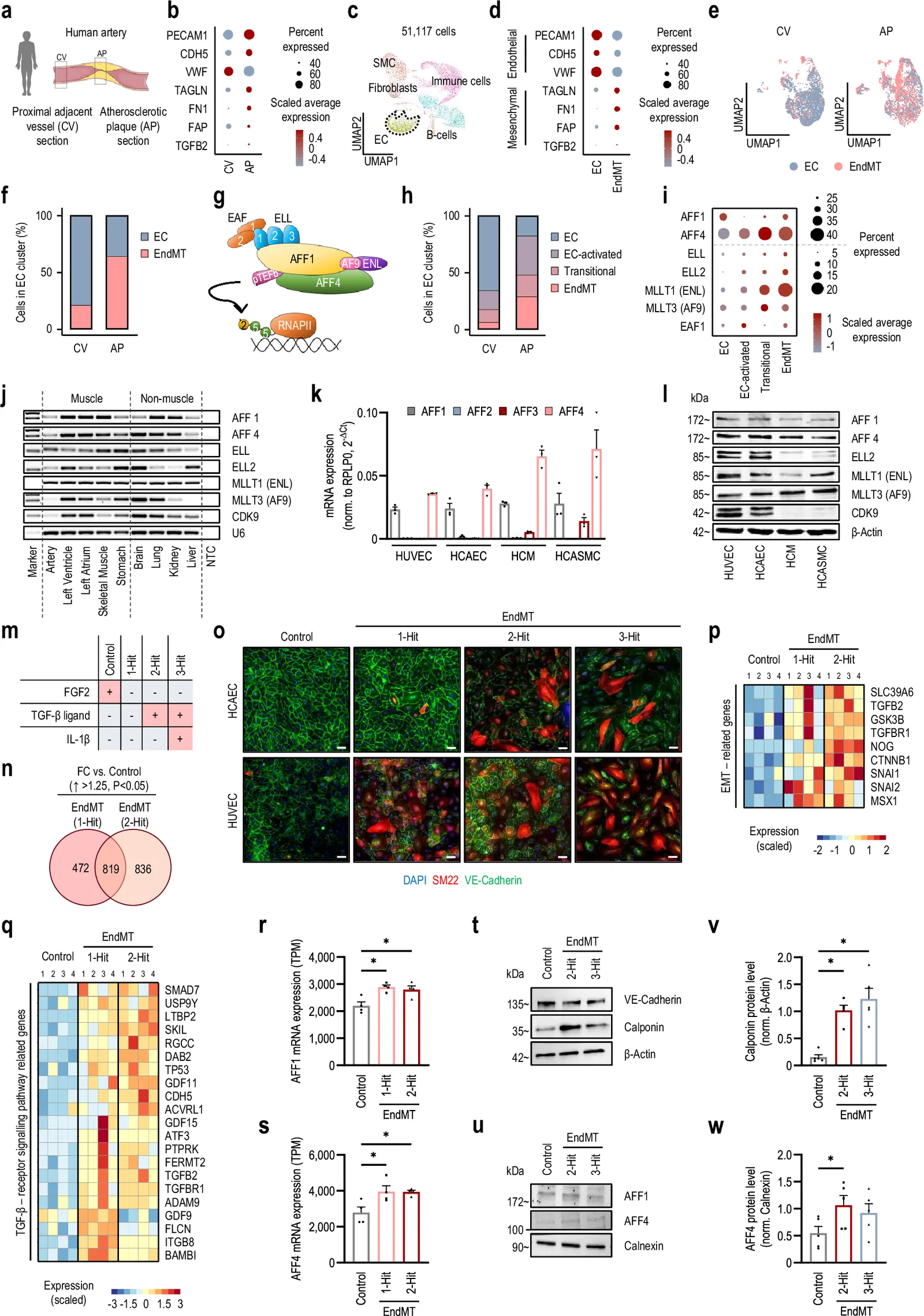
The SEC scaffold proteins AFF1 and AFF4 are expressed in human endothelial cells (ECs) and upregulated during the endothelial-to-mesenchymal transition (EndMT). **a** Single-cell sequencing data of human carotid artery plaques and proximal adjacent vessel sections used as control from the same patients (*n* = 3 per group). **b** Expression of EndMT signature genes in single-cell sequencing analysis from AP and CV tissue depicted in a dot plot (*n* = 3 per group). **c** Cell clustering of 51,117 cells from atherosclerotic plaque and control sections depicted in a UMAP plot. **d** Re-clustering of the EC cluster into EndMT-positive and EndMT-negative ECs in atherosclerotic plaques based on expression of EC markers *PECAM1, CDH5, VWF*, and EndMT markers *TAGLN, FN1, FAP*, and *TGFB2* (depicted by a dot plot) was used as an EndMT signature score (n = 3 per group). **e** EndMT-positive and EndMT-negative ECs in atherosclerotic plaques (AP) (right) and proximal adjacent vessels section used as control (CV) (left). **f** Quantitative analysis of the mean percentage of EC (EndMT^neg^) and EndMT-positive cells in the EC cluster of AP and CV. **g** Schematic depiction of super elongation complex (SEC) composition built around the scaffold proteins AFF1 and AFF4. (**h**) Quantitative analysis of the mean percentage of quiescent EC, EC-activated, Transitional, and EndMT-positive cells in the EC cluster of AP and CV. (**i**–**l**) Expression of the SEC genes in ECs of human plaques and different human tissues and cardiovascular cell types. **i** SEC-associated gene expression in ECs (EndMT^neg^), EC-activated, Transitional, and EndMT-positive cells of carotid artery plaques and control vessel tissue depicted in a dot plot. **j** mRNA Expression of SEC-component genes in different human tissues as determined by semi-quantitative PCR. **k** AFF1-AFF4 expression in human umbilical vein endothelial cells (HUVECs), human carotid artery endothelial cells (HCAECs), human coronary artery smooth muscle cells (HCASMCs) and human cardiomyocytes (HCMs) determined by qPCR (n = 3). (**l**) Protein expression of SEC components in different human cardiovascular cell types. **m** EndMT in human ECs using a multi-hit strategy. **n** HCAECs were subjected to a 1-Hit (-FGF-2) or 2-Hit (-FGF-2/+TGF-β2) EndMT model, and gene expression was analyzed using bulk RNA sequencing (*n* = 4 per group). Venn diagram depicting upregulated genes in human coronary ECs undergoing EndMT. **o** Representative immunofluorescence staining of ECs under EndMT conditions with SM22 (red) and VE-Cadherin (green) and DAPI counterstain (blue) detected at 32X magnification. Scale bar represents 20 µm. Heatmaps depicting the expression of **p** EMT-related and **q** TGF-β receptor signaling pathway-related genes in human coronary ECs undergoing 1-Hit and 2-Hit EndMT vs. control ECs using RNA-seq (*n* = 4 per group). **r** AFF1 and **s** AFF4 expression in human coronary ECs undergoing EndMT (*n* = 4, One-way ANOVA, Dunnett’s multiple comparison test) as TPM using RNA-seq (*n* = 4/group). **t** Protein expression of EndMT-associated genes and **u** SEC components AFF1 and AFF4 in human coronary ECs (*n* = 4). **v** Quantification of Calponin and **w** AFF4 protein levels in EndMT-treated human coronary ECs (n = 5, One-way ANOVA, Dunnett’s multiple comparison test). CV – control vessel, AP – atherosclerotic plaque, Ctl – control. All bar graphs represent mean + SEM, * indicates *P* < 0.05

Atherosclerotic plaque and proximal adjacent vessel sections used as controls from the same patients were analyzed by single-cell RNA sequencing (scRNA-seq) (Fig. 1a–h). Atherosclerotic plaque samples showed increased mesenchymal markers such as *TAGLN, FN1, FAP*, and *TGFB2*, indicating EndMT-positive ECs (Fig. 1b). After cell clustering, the EC cluster was separated into EndMT-positive and EndMT-negative EC based on single-cell expression of EC and mesenchymal marker gene expression (Fig. 1c–f). The number of EndMT-positive ECs was increased in atherosclerotic plaque sections compared to control (proximal adjacent) vessel sections (Fig. 1e, f). Further separation of the EC transition process into an early EC-activated, a transitional signature, and a late EndMT signature showed components of the SEC activation as a hallmark of the transitioning endothelial state in human plaques (Fig. 1g–i). Expression of the SEC scaffolds AFF1 and AFF4 was reduced in fibroblasts, immune cells and smooth muscle cells of atherosclerotic plaque sections compared to control (proximal adjacent) vessel sections (Supplementary Fig. 1b). All SEC components were expressed in human cardiovascular tissues, such as left ventricle and atrium and in primary ECs isolated from human coronaries (Fig. 1j–l, Supplementary Fig. 1c). In human coronary artery ECs, EndMT was induced using 1-, 2- and 3-hit models that combine FGF2 loss, TGF-β and IL-1β treatment. This induction increased levels of SEC complex components at both the RNA and protein levels and was accompanied by a concomitant onset of endothelial dysfunction (Fig. 1m–w, Supplementary Fig. 1d–m).^6^ Together, our data indicate that SEC complex components are increased in EndMT-positive ECs of human atherosclerotic plaques.

### Pharmacologic inhibition of RNA elongation via SEC reduces EndMT induction

**Fig. 2:**
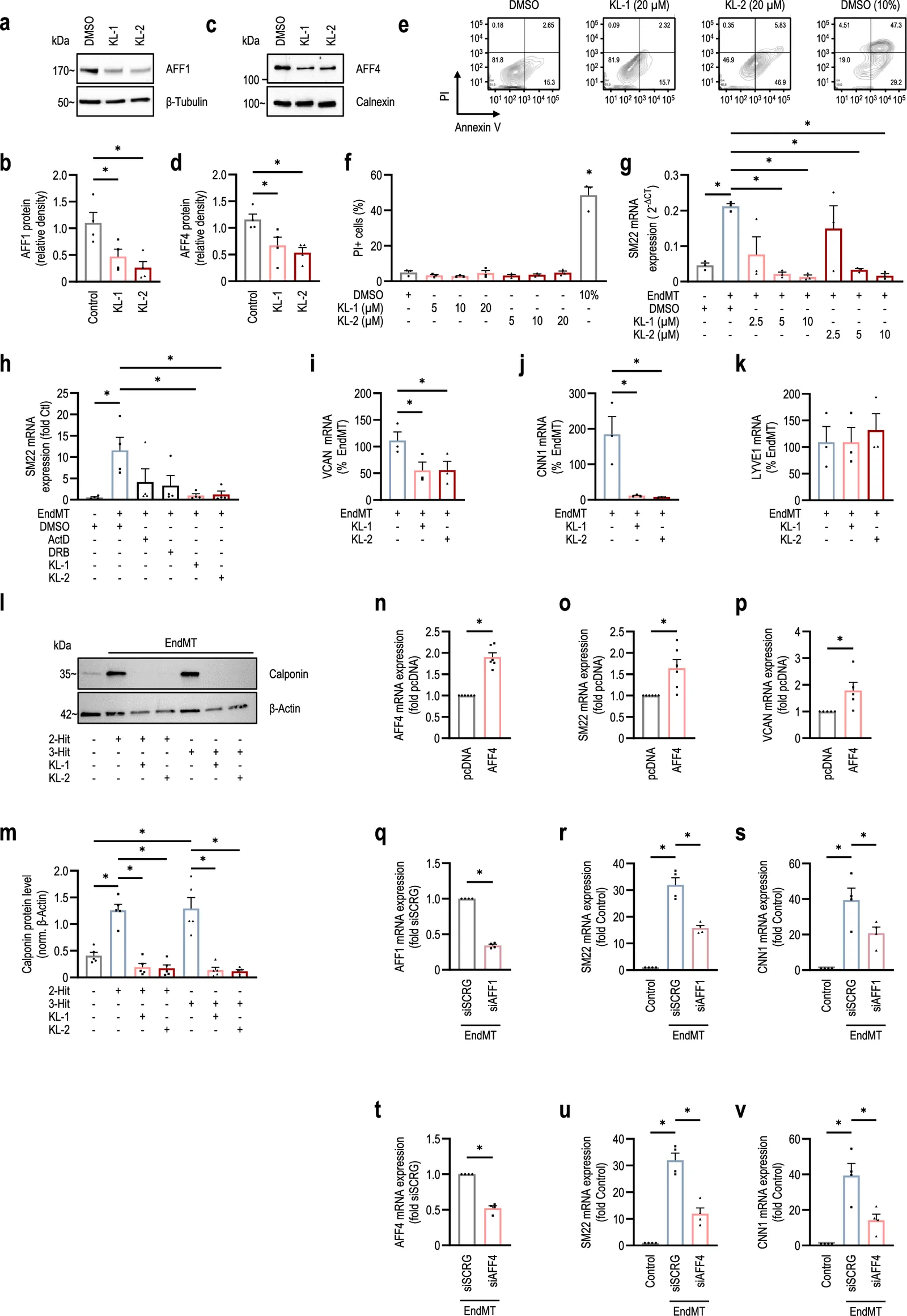
SEC inhibition with small molecules reduces EndMT induction in human ECs. **a** Representative immunoblots and **b** quantification of AFF1 (n = 4, one-way ANOVA with Tukey’s multiple comparisons test). **c** Immunoblot and **d** quantification of AFF4 protein expression in ECs treated with 20 µM of SEC inhibitors KL-1 and KL-2 for 6 h (*n* = 4, One-way ANOVA, Tukey correction) **e** Propidium iodide-positive ECs after treatment with increasing concentrations of KL-1 and KL-2 for 24 hours determined by flow cytometry. 10% DMSO serves as a positive control (*n* = 3). **f** Quantification of Propidium iodide-positive ECs (*n* = 3, One-way ANOVA, Tukey correction). **g** *SM22* mRNA expression in ECs undergoing EndMT treated with increasing concentrations of KL-1 and KL-2 (2.5, 5 or 10 µM) for 72 hours determined by qPCR (*n* = 3, one-way ANOVA, Tukey correction). **h** *SM22* mRNA expression in ECs undergoing EndMT treated with different transcription inhibitors: Actinomycin D (1 µg/mL), DRB (100 µM), KL-1 (5 µM), KL-2 (5 µM) for 72 hours determined by qPCR (*n* = 4, One-way ANOVA, Tukey correction). Expression of **i** *VCAN* **j** *CNN1* (*n* = 3, One-way ANOVA with Dunnett’s multiple comparisons test), and **k** *LYVE1* mRNA expression in ECs undergoing EndMT treated with 5 µM KL-1 or KL-2 for 72 hours (*n* = 3, Kruskal-Wallis test with Dunn’s multiple comparisons test). **l** Immunoblot depicting β-Actin and Calponin protein levels in human coronary ECs under control and EndMT conditions with or without SEC inhibitor treatment and **m** quantification of Calponin protein levels normalized to β-Actin (*n* = 5, One-way ANOVA, Tukey correction). Quantification of **n** AFF4, **o** SM22 and **p** VCAN mRNA expression using qRT-PCR after 72 hours AFF4 overexpression compared to pcDNA control (*n* = 5-6, t-test). The levels of AFF1, AFF4, SM22, and CNN were determined by qRT-PCR 24 h after knockdown of **q**–**s** AFF1 or **t**–**v** AFF4 and 72 h EndMT (*n* = 4, q/t: t-test; r/s/u/v: One-way ANOVA, Tukey correction). ActD – actinomycin D, PI – propidium iodide, Ctl – control, VE-Cad. – VE-Cadherin. EndMT indicates the 2-Hit EndMT model. All bar graphs represent mean + SEM, * indicates *P* < 0.05. Biological replicates indicated by n, technical replicates were averaged within each biological replicate

Components of the RNA elongation complex are induced in ECs undergoing mesenchymal transition in human atherosclerotic plaques. Therefore, we assessed a potential effect of these two SEC component proteins on the process of EndMT. Treatment of human coronary ECs using two small-molecule inhibitors (KL-1 and KL-2) for 6 hours already reduced AFF1 and AFF4 protein levels (Fig. 2a–d). Treatment with increasing concentrations of SEC inhibitors did not induce cell death in ECs, as determined by Annexin V and Propidium Iodide staining followed by flow cytometry analysis (Fig. 2e, f, supplementary Fig. 2). Inhibition of both RNA transcription and RNA elongation using actinomycin D and 5,6-dichlorobenzimidazole 1-β-D-ribofuranoside (DRB) during EndMT induction inhibited the increase of mesenchymal marker SM22 (Fig. 2g, h). Treatment of human ECs using KL-1 and KL-2 inhibited EndMT induction, as indicated by mesenchymal marker gene expression, including SM22 (*TAGLN*), versican (*VCAN*) and calponin (*CNN1*), while endothelial marker expression was not affected (Fig. 2g–m). Overexpression of AFF4 in primary human ECs induced mesenchymal marker gene expression, including SM22 (*TAGLN*) and versican (*VCAN*) even without additional EndMT stimuli (Fig. 2n–p). Knockdown of AFF1 or AFF4 during EndMT reduced markers of mesenchymal transition (Fig. 2q–v). Taken together, our data show that inhibition of SEC-mediated RNA elongation can reduce the induction of EndMT.

### EndMT induction results in genome-wide release of paused RNAPII from proximal promoters via SEC

**Fig. 3:**
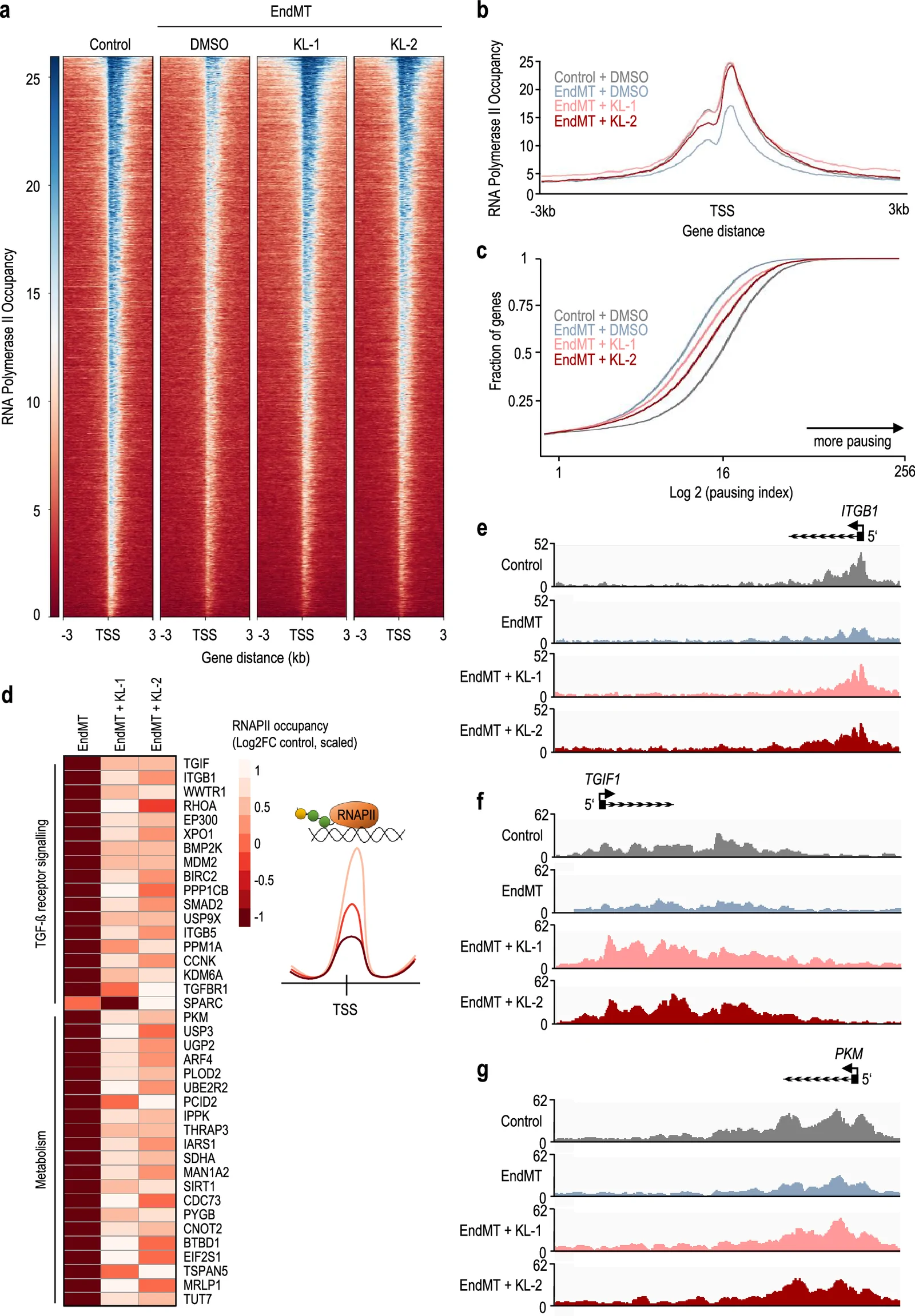
The process of EndMT in human ECs is controlled by RNA elongation and can be inhibited by SEC inhibitor treatment. **a** Heatmap of promoter-proximal RNA Polymerase II (RNAPII) peaks in ECs treated with KL-1 and KL-2 under control and EndMT-induction conditions. Promoter-proximal RNAPII occupancy is depicted 3 kb up- and downstream of the TSS. **b** Histogram of RNAPII occupancy proximal to the TSS and **c** pausing index of RNAPII in EndMT and cultured ECs treated with SEC inhibitors calculated with SICER. **d** Heatmap showing fold changes of RNAPII occupancy in promoter proximity of genes associated with TGF-β receptor signaling and metabolism. Gene tracks extracted from the Integrative Genome Viewer (IGV) from SEC-regulated genes **e** *ITGB1*, **f** *TGIF1*, and **g** *PKM* in ECs treated with SEC inhibitors under control and EndMT conditions. Kb – kilobases, TSS – transcriptional start site. EndMT indicates the 2-Hit EndMT model. Biological replicates indicated by *n*, technical replicates were averaged within each biological replicate

The transition from RNA initiation to active RNA elongation is controlled by the SEC through the release of RNAPII from promoter-proximal pausing. We observed that inhibition of the SEC reduces EndMT and investigated the genome-wide RNAPII occupancy after the induction of EndMT. RNAPII occupancy in the proximal regions of the promoter decreased after EndMT induction, indicating a release of paused RNAPII towards active elongation after EndMT induction as analyzed by chromatin sequencing after immunoprecipitation of RNAPII (Fig. 3a). EndMT induction resulted in a lower RNAPII occupancy proximal to the transcriptional start site, which was reverted by SEC inhibition (Fig. 3b). These data suggest that RNAPII was stalled in ECs under control conditions but shifted into active transcription during the induction of EndMT, indicating a dynamic change in promoter-proximal pausing. Indeed, a calculation of the pausing index revealed less promoter-proximal pausing of RNAPII in ECs undergoing EndMT than in ECs under control conditions. Treatment of ECs with SEC inhibitors could again increase the pausing indices after induction of EndMT (Fig. 3c). Gene ontology analysis of the genes with changes in RNAPII occupancy at the promoter sites revealed EndMT-related induction of TGF-β-associated mesenchymal as well as metabolic gene induction (Fig. 3d–g).

Taken together, our data suggest that the induction of EndMT leads to genome-wide changes in RNAPII states and results in reduced promoter-proximal pausing, which indicates a regulatory contribution of the SEC-controlled RNA elongation during the onset of EndMT.

### RNA elongation rates increase within minutes after induction of EndMT and can be inhibited by pharmacologic intervention

As our data revealed global changes in RNAPII promoter-proximal pausing, we hypothesized that EndMT induction is linked to increased RNA elongation rates in ECs. To analyze RNA elongation rates, we performed 4sU-DRB sequencing. We pre-treated ECs with SEC inhibitors KL-1 and KL-2, induced EndMT, and transiently inhibited RNA elongation with DRB. After inhibitor washout, 4-thiouridine (4sU) was added to the ECs and incorporated into newly transcribed RNA. After a biotin-IP, 4sU-labelled RNA was analyzed using next-generation sequencing.

**Fig. 4:**
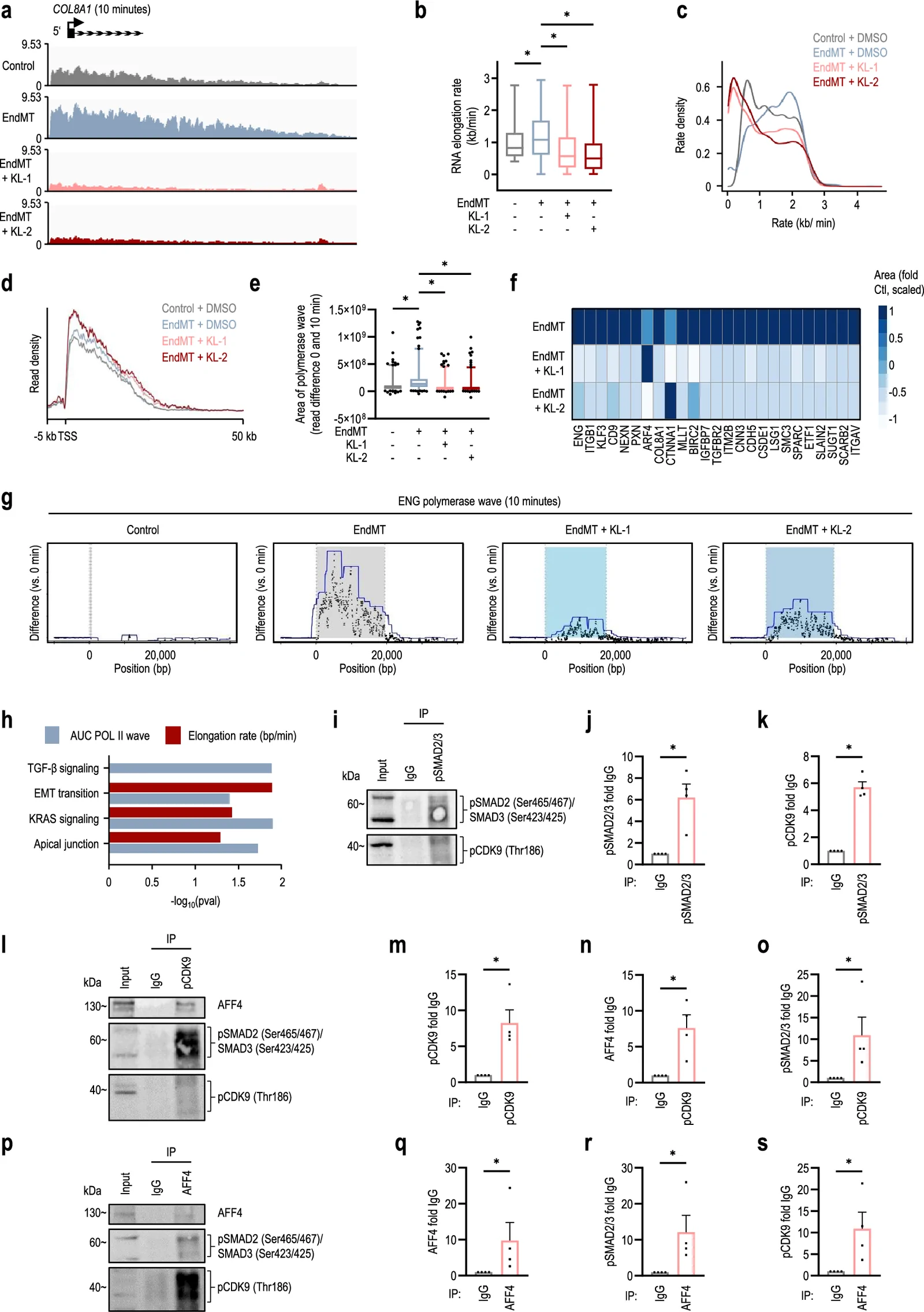
Increase in RNA elongation rates during the early induction of EndMT in human endothelial cells. **a** Gene tracks extracted from the IGV of *COL8A1*. **b** Box-whisker plot depicting RNA elongation rates calculated by SICER of SEC-mediated genes (*n* = 934 genes per group, Friedman test with Dunn’s multiple comparisons test). **c** Histograms of distribution of RNA elongation rates in all tested genes in ECs with or without EndMT and KL-1/ KL-2 treatment. **d** Average read densities over the gene body depicted in ECs undergoing EndMT and SEC inhibition. **e** Box-whisker plot depicting the area under the polymerase wave calculated with groHMM (*n* = 934 genes, Friedman test with Dunn’s multiple comparisons test). **f** Heatmap showing fold changes of calculated areas under the polymerase wave. Polymerase wave of **g** *ENG* calculated using the Hidden Markov model with groHMM depicting the read changes between 10 and 0 minutes after EndMT induction with or without KL-1 and KL-2 inhibition. **h** Bar plot showing significantly enriched Hallmark gene sets identified by gene set enrichment analysis (GSEA) based on 4sU-seq data. Genes were ranked according to the log₂ fold change between EndMT and control conditions. Two independent peak quantification approaches were used: area of the polymerase II wave (calculated by groHMM; blue) and elongation rate (calculated by SICER; red). Pathways detected by both approaches are displayed as adjacent bars. Only pathways with *p* < 0.05 are shown (Ranked-GSEA). Co-immunoprecipitation of pSMAD2/3, pCDK9 and AFF4 (**i**, **l**, **p**) validated and (**j**, **m**, **q**) quantified using immunoblotting (n = 4). Quantification of interaction partners (**k**, **s**) pCDK9, (**n**) AFF4 and (**o**, **r**) pSMAD2/3 (*n* = 4, j/n/o/q/r/s: t-test; k/m: Mann-Whitney test). synth. – synthesized, 4sU – 4-thiouridine, * indicates *P* < 0.05. EndMT indicates the 2-Hit EndMT model. Biological replicates indicated by *n*, technical replicates were averaged within each biological replicate

EndMT induction and SEC inhibition using KL-1 and KL-2 markedly altered RNA elongation rates, as seen on the gene tracks of the EndMT-associated gene *COL8A1* (Fig. 4a) and genome-wide analysis of the RNA elongation rate. The mean RNA elongation rate in ECs was 0.98 kb/min. The induction of EndMT led to a 19.4% increase in mean RNA elongation rate, while inhibition (KL-1) of the SEC reduced RNA elongation rates during EndMT by 34.2% (KL-2: 43.6%, Fig. 4b). The changes in RNA elongation rates were also visible in the density distribution of RNA elongation rates (Fig. 4c) and in the mean read densities over the whole gene body of regulated transcripts (Fig. 4d). Untreated ECs had a higher count of genes with elongation rates lower than 1 kb/min than cells undergoing EndMT. However, induction of EndMT led to a “right-shift” with a higher count of genes with RNA elongation rates higher than 2 kb/min (Fig. 4c). However, there was no global decrease in RNA elongation rates after treatment with KL-1 or KL-2, indicating EndMT-selective regulation. To capture immediate changes in RNA initiation and RNA elongation, we calculated the area under the transcriptome-wide polymerase wave showing an overall increase after induction of EndMT (Fig. 4e). Genes related to TGF-β signaling and markers of EndMT such as *ENG, ITGB1* or *TGFBR2* showed the largest increase in the area under the polymerase wave analysis (Fig. 4f). Induction of EndMT increased RNA initiation and the polymerase wave of *COL8A1* and *ENG*, and both were reduced by treatment with SEC inhibitors (Fig. 4g, Supplementary Fig. 3). The SEC-induced polymerase wave, which started at the 5’ start of the genes, was already increased 10 minutes after the induction of EndMT (Fig. 4g, Supplementary Fig. 3). Gene Set Enrichment Analysis (GSEA) of the 4sU-DRB-seq elongation rate data and the area under the RNA polymerase II wave revealed significant enrichment of TGF-β signaling and epithelial to mesenchymal transition (EMT) among SEC-regulated elongation targets (Fig. 4h). To directly address whether SEC integrates with the canonical EndMT signaling pathway, we performed reciprocal nuclear co-immunoprecipitation (co-IP) experiments in TGF-β2-stimulated primary human ECs during EndMT. AFF4 immunoprecipitation enriched both pCDK9(T186) and pSMAD2/3. Reciprocal pSMAD2/3 immunoprecipitation enriched AFF4 and pCDK9, and pCDK9 immunoprecipitation similarly enriched AFF4 and pSMAD2/3. These reciprocal interactions establish that activated SMAD2/3 — the canonical EndMT transcription factor — physically associates with the activated SEC scaffold/kinase complex during TGF-β-driven EndMT (Fig. 4i–s). Taken together, our findings suggest that RNA elongation is increased by the induction of EndMT, which leads to a rapid increase in the expression of regulatory genes and genes associated with EndMT and is reduced by pharmacological inhibition.

### Pharmacological SEC inhibition reduces EndMT-induced cardiac functional impairment and collagen deposition in a functional human cardiac organoid model

**Fig. 5:**
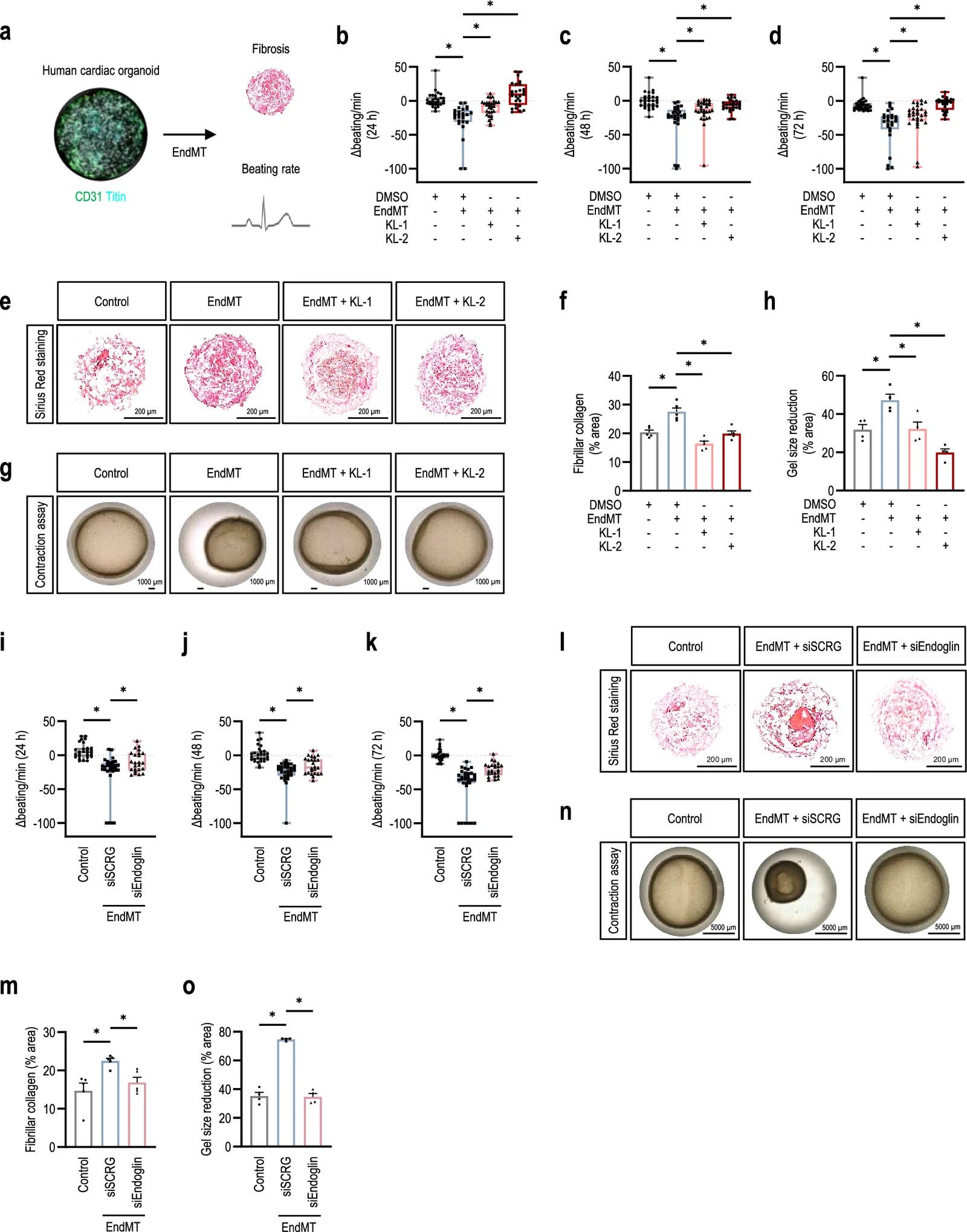
Pharmacological SEC inhibition reduces EndMT-induced cardiac functional impairment and collagen deposition in a functional human cardiac organoid model. **a** Overview of experimental workflow: analysis of fibrosis in human cardiac organoids after EndMT using sirius-red staining and beating rate. Quantification of the beating rate (beats/min) of human cardiac organoids cultured in control medium (DMSO), EndMT medium (DMSO), or EndMT medium supplemented with KL-1 (5 µM) or KL-2 (5 µM) after **b** 24 h, **c** 48 h, and **d** 72 h of EndMT treatment (n = 29 (Control), n = 26 (EndMT + DMSO), n = 30 (EndMT + KL-1), *n* = 27 (EndMT + KL-2), one-way ANOVA, Dunnett’s multiple comparisons test). **e**, **f** Quantification of the percentage of fibrillar collagen area in human cardiac organoids under the same conditions using sirius-red staining (*n* = 5, one-way ANOVA, Dunnett’s multiple comparisons test). **g**, **h** Contractility of human coronary artery endothelial cells (HCAECs) cultured in control medium (DMSO), EndMT medium (DMSO), or EndMT medium supplemented with KL-1 (5 µM) or KL-2 (5 µM), assessed by collagen gel contraction assay and quantified as gel size reduction (n = 4, one-way ANOVA, Dunnett’s multiple comparisons test). Quantification of the beating rate (beats/min) of human cardiac organoids in control medium or after knockdown with control siRNA or siEndoglin prior to EndMT treatment, measured after **i** 24 h, **j** 48 h, and **k** 72 h (*n* = 28 (control), *n* = 32 (SiScrG), *n* = 24 (SiEndoglin), one-way ANOVA, Dunnett’s multiple comparisons test). **l**, **m** Quantification of the percentage of fibrillar collagen area in human cardiac organoids in control medium or after knockdown with control siRNA or siEndoglin prior to EndMT treatment, using sirius-red staining (n = 5, one-way ANOVA, Dunnett’s multiple comparisons test). **n**, **o** Contractility of HCAECs in control medium or after knockdown with control siRNA or siEndoglin prior to EndMT treatment, assessed by collagen gel contraction assay and quantified as gel size reduction (*n* = 4, one-way ANOVA, Dunnett’s multiple comparisons test)

We developed a functional human cardiac organoid model with consistent beating, which for the first time enables assessment of the impact of EndMT on cardiac function in vitro. The EndMT organoid model results in increased fibrosis accompanied by enhanced ECM deposition and reduced cardiac beating rates (Fig. 5a). Treatment of organoids with KL-1 or KL-2 rescued the EndMT-induced reduction in cardiac beating (Fig. 5b–d). Accordingly, KL-1 or KL-2 treatment reduced fibrotic transition indicated by reduced ECM deposition (Fig. 5e, f). We further developed a model for ECs undergoing EndMT acquiring fibrotic contractile properties in a matrix gel assay. Treatment with KL-1 or KL-2 reduced fibrotic contractility during EndMT-induction (Fig. 5g, h). The reduction of the top target endoglin (*ENG*) identified by RNA elongation rate analysis during EndMT recovered EndMT-induced reduction in cardiac beating (Fig. 5i–k). In line, knockdown of *ENG* reduced fibrotic transition in the cardiac organoid model indicated by reduced ECM deposition (Fig. 5l, m). Reduced *ENG* level prevented fibrotic contractility during EndMT-induction (Fig. 5n, o). This represents the first demonstration that EndMT in the endothelial compartment compromises cardiomyocyte function in a non-cell-autonomous manner, and that this organ-level dysfunction is reversed by SEC inhibition. These three readouts — matrix production, contractility, and impact on cardiomyocyte function — together establish robust functional consequences of EndMT and their reversibility by both pharmacological and genetic SEC pathway inhibition.

### Pharmacological SEC inhibition reduces atherosclerotic plaque formation and immune cell infiltration and reduces features of plaque vulnerability in a preventive and a therapeutic atherosclerosis mouse model

**Fig. 6:**
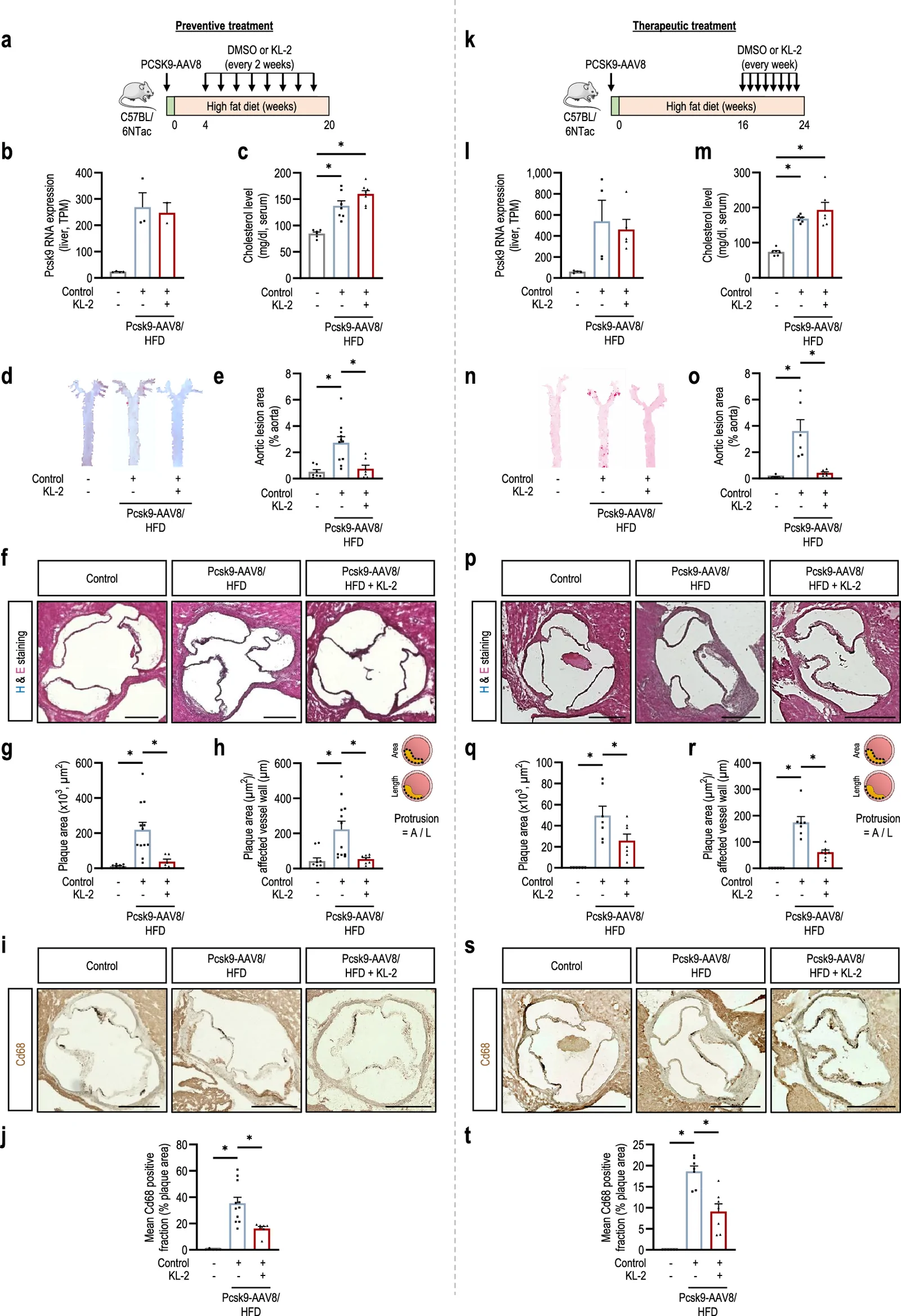
Pharmacological SEC inhibition reduces atherosclerotic plaque formation and immune cell infiltration and reduces features of plaque vulnerability in a preventive and a therapeutic atherosclerosis mouse model. **a**–**j**, **k**–**t**) The effect of SEC inhibition (using KL-2) was analyzed in vivo using a hyperlipidemia-induced (high-fat diet (HFD)) atherosclerosis mouse model without genetic knockout of metabolic genes (*Pcsk9*-D377Y-AAV8 with liver-specific promoter). Animals received KL-2 treatments either preventively (**a**–**j**) or in (**k**–**t**) therapeutically mouse model of atherosclerosis. **a**/**k:** **a** *Pcsk9*-AAV8 was injected into the tail vein of C57BL/6NTac mice, after 1-week mice were subjected to a high-fat diet (HFD). After four weeks, the levels of cholesterol in the serum were analysed, and the mice were randomly and blindly selected for injection with either KL-2 (10 mg/kg BW) or the corresponding volume of DMSO, at fortnightly intervals until the end of the 20-week experiment. (**k**) *Pcsk9*-AAV8 was injected into the tail vein of C57BL/6NTac mice, after 1-week mice were subjected to a high-fat diet (HFD). At 16 weeks, the mice were randomly and blindly selected for injection with either KL-2 (10 mg/kg BW) or the corresponding volume of DMSO, at weekly intervals until the end of the 24-week experiment. (**b**/**l**) *Pcsk9* mRNA expression in livers of mice treated with *Pcsk9*-AAV8 (**b**: *n* = 4 (DMSO) vs. *n* = 3 (KL-2); **l**: *n* = 4 (DMSO) vs. 5 (KL-2)) or PBS after (**b**) 20 weeks or (**l**) 24 weeks as analyzed by RNA seq. **c**/**m** Cholesterol serum levels at **c** 20 weeks (*n* = 6–8 per group, one-way ANOVA, Dunnett’s multiple comparisons test), or (**m**) 24 weeks (*n* = 6 per group, Kruskal-Wallis test with Dunn’s multiple comparisons test). **d**/**n** Representative depictions and (**e**/**o**) quantification of en face Oil Red O stainings of mice aortae (**e**: *n* = 7–11 per group, One-way ANOVA, Dunnett’s multiple comparisons test; **o**: *n* = 6-7 per group, Kruskal-Wallis test with Dunn’s multiple comparisons test). **f**/**p** Representative H & E stainings performed on aortic root sections and (**g**/**q**) quantification of plaque area. (**g**: *n* = 6–12 per group, One-way ANOVA, Dunnett’s multiple comparison test; **q**: *n* = 6-7 per group, one-way ANOVA, Dunnett’s multiple comparisons test). **h**/**r** Reduction in features of plaque vulnerability indicated by plaque protrusion into the vascular lumen (**h**: *n* = 8–12 per group, Kruskal-Wallis test with Dunn’s multiple comparisons test; **r**: *n* = 6–7 per group, Kruskal-Wallis test with Dunn’s multiple comparisons test). **i**/**s** Representative depictions of CD68-positive cells of atherosclerotic plaques in aortic root sections of mice. Quantification of CD68-postive area in atherosclerotic plaques (**j**: *n* = 5–11 per group, Kruskal-Wallis test with Dunn’s multiple comparisons test; **t**: *n* = 6–7 per group, Kruskal-Wallis test with Dunn’s multiple comparisons test). NC – Normal Chow, HFD – High Fat Diet. All bar graphs represent mean + SEM, * indicates *P* < 0.05. AP – atherosclerotic plaque; M – media. Biological replicates indicated by *n*, technical replicates were averaged within each biological replicate

We identified the SEC as a regulator during the onset of EndMT, as RNAPII pausing was reduced and RNA elongation increased during EndMT, and an inhibition of the SEC led to reduced expression of mesenchymal marker genes. The effect of SEC inhibition (using KL-2 based on its higher efficiency) was analyzed in vivo in a preventive and a therapeutic treatment setup using a hyperlipidemia-induced (high-fat diet (HFD)) atherosclerosis mouse model without genetic knockout of metabolic genes (*Pcsk9*-D377Y-AAV8 with liver-specific promoter). In the preventive model setup, fortnightly KL-2 treatment began 4 weeks after the start of the high-fat diet (5 weeks after *Pcsk9*-D377Y-AAV8) (Fig. 6a–j), whereas in the therapeutic setup, weekly KL-2 treatment began 16 weeks after the start of the high-fat diet (Fig. 6k–t). In both models, treatment with the KL-2 inhibitor did not affect the increase in serum cholesterol levels in the atherosclerotic group (Fig. 6c, m). Inhibitor treatment did not affect the systemic level of inflammatory cytokines Il-1β, Il-6, Tnf, or Ifn-γ or the expression of inflammation markers Il-1b (*Il1b*), Il-6 (*Il6*), or *Tnf* in circulating immune cells (Supplementary Fig. 4a–g). However, the expression of EndMT target genes controlled by RNA elongation (Fig. 4f) was reduced in mouse tissue after RNA elongation was inhibited (Supplementary Fig. 4j–m). Pharmacologic inhibition of RNA elongation showed reduced plaque burden in the aorta in both models compared to control mice (Fig. 6d, e, n, o). KL-2 inhibitor treatment in both models further reduced overall plaque burden (Fig. 6f, g, p, q) and immune cell infiltration in the aortic root (Fig. 6i, j, s, t) but, more importantly, reduced features of plaque instability as indicated by plaque protrusion (Fig. 6h, r).

### SEC inhibition reduces TGF-β signaling and features of plaque vulnerability in a preventive and a therapeutic atherosclerosis mouse model in vivo

**Fig. 7:**
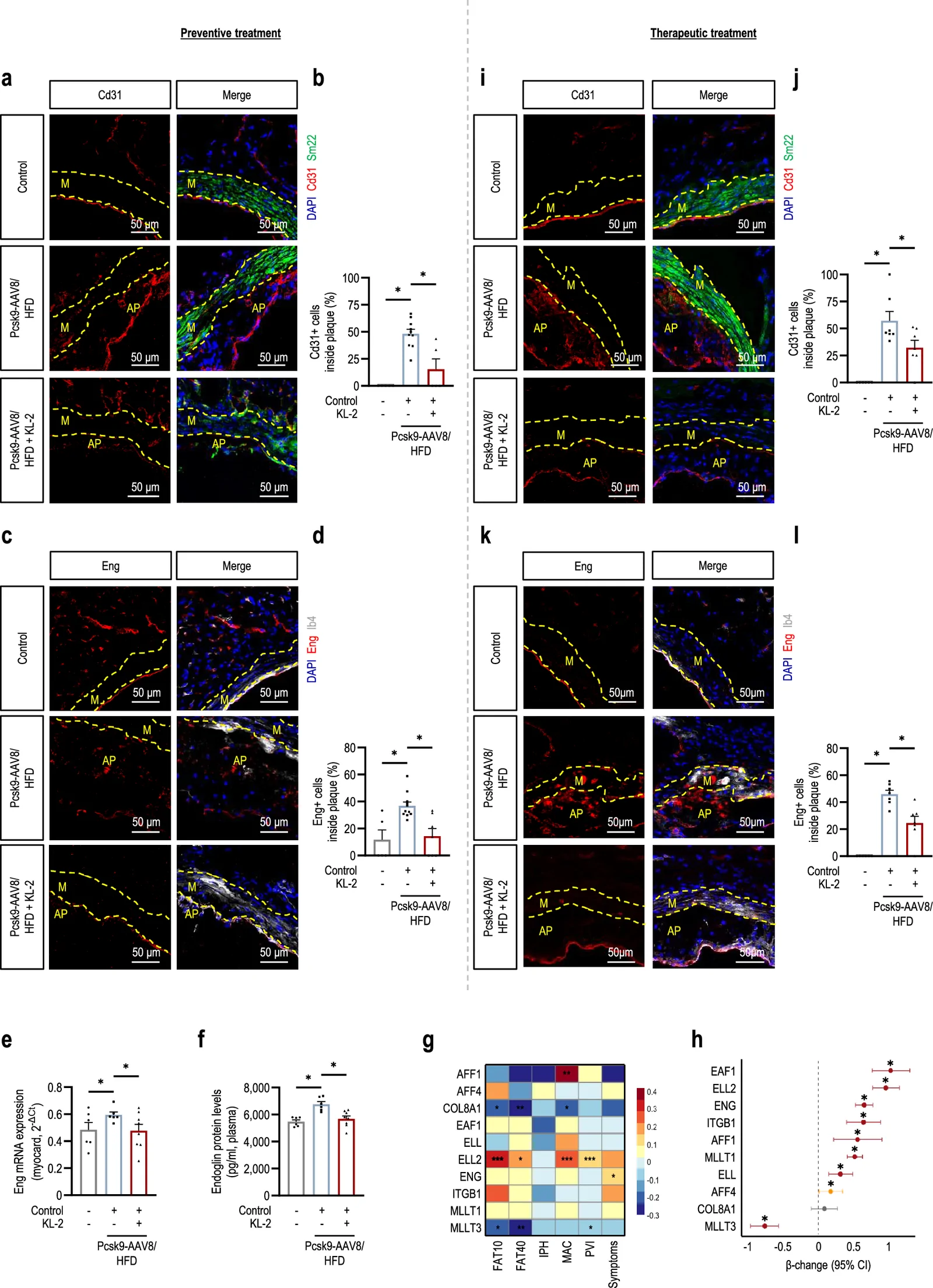
Analysis of EndMT and RNA elongation in atherosclerotic plaques of mice and humans. **a**/**i** Representative depiction of immunofluorescence staining of Cd31 (red) and DAPI (blue), Cd31 (red) and Sm22 (green) in the merge picture in aortic root sections and quantification of CD31 positive cells inside atherosclerotic plaques (**b**: *n* = 5–10 per group, Kruskal-Wallis test with Dunn’s multiple comparisons test; **j**: n = 6-7 per group, one-way ANOVA, Dunnett’s multiple comparisons test). **c**/**k** Representative depiction of immunofluorescence staining of Endoglin CD105 (red) and DAPI (blue), Endoglin (red) and Ib4 (white) in the merge picture in aortic root sections and **d**/**l** quantification of Endoglin/Ib4 positive cells in atherosclerotic plaques (**d**: *n* = 5–11 per group, Kruskal-Wallis test with Dunn’s multiple comparisons test; **l**: *n* = 6–7 per group, one-way ANOVA, Dunnett’s multiple comparisons test). **e** Endoglin mRNA expression in myocardial tissue of mice measured by qPCR (*n* = 6–8, t-test). **f** Endoglin plasma protein levels of atherosclerotic mice with or without KL-2 treatment determined by ELISA (n = 6-8, One-way ANOVA, Dunnett’s multiple comparisons test). **g** Associations of human plaque characteristics (FAT10, FAT40, IPH, MAC, PVI, Symptoms). Associations between gene expression and clinical symptoms, plaque phenotype, and histological features were assessed using generalized linear models, while the association with PVI was evaluated using Spearman’s rank correlation coefficient. **h** Vulnerable vs. stable atherosclerotic plaques of 1,048 human samples with RNA elongation process. Data in (**h**) were corrected for age and sex. To correct for multiple testing, Benjamini-Hochberg (BH) adjustment was applied, with an FDR < 0.05 considered significant (red dots in (**h**)). * indicates *P* < 0.05. Biological replicates indicated by *n*, technical replicates were averaged within each biological replicate. AP – atherosclerotic plaque; M – media

We found SEC inhibitor treatment to reduce EndMT induction in human ECs. Therefore, we aimed to analyze the effect of SEC inhibitor treatment on EndMT and plaque stability in a preventive and a therapeutic hyperlipidemia-induced atherosclerosis mouse model in vivo. EndMT, characterized by various markers such as the signaling molecules TGF-β1 and TGF-β2, TGF-β target gene expression such as *Cdh2* as well as cellular markers of EndMT such as IB4- or CD31-positive cells within atherosclerotic plaques, as well as CD31/SM22 double-positive cells, was quantified in atherosclerotic plaques in the aortic root (Fig. 7a–d, i–l, Supplementary Fig.5a, b and 6a–f).^28^ The number of EndMT-positive ECs inside atherosclerotic plaques, as a measure of plaque instability, decreased in both atherosclerotic mice models after treatment with the RNA elongation inhibitor KL-2 (Fig. 7a, b, i, j, Supplementary Fig. 5a, b). In both mice models, treatment with RNA elongation inhibitor KL-2 further reduced TGF-β signaling and target gene expression within atherosclerotic plaques (Supplementary Fig. 6a–f). Endoglin was the top regulated gene at the RNA elongation level during EndMT and was reduced by SEC inhibitor treatment (Fig. 4f, g). The number of *ENG*-positive cells inside atherosclerotic plaques decreased in both atherosclerotic mice models after treatment with the RNA elongation inhibitor KL-2 (Fig. 7c, d, k, l). Transcriptional analysis of myocardial tissue from atherosclerotic mice after RNA elongation inhibitor treatment showed reduced *Eng* expression (Fig. 7e), while protein analyses demonstrated reduced circulating soluble endoglin concentrations (Fig. 7f). Finally, analysis from 1,048 human atherosclerotic plaque segments from the Athero-Express biobank showed associations of expression levels of core SEC/Elongation axis genes with multiple histological plaque features, including macrophage content (MAC), intraplaque hemorrhage (IPH), fat content (>10% or >40%), presence of severe symptoms (TIA and stroke), and for plaque vulnerability index score (PVI), even after correction for age and sex (Fig. 7g). Vulnerable molecular plaque types^29^ showed increased expression of RNA elongation components and RNA elongation targets of EndMT such as *ITGB1* and *ENG* (Fig. 7h). Taken together, our data indicate that inhibiting RNA elongation has atheroprotective effects by reducing plaque burden, but more importantly reducing features associated with plaque vulnerability, a major risk factor for myocardial infarction and stroke, through early inhibition of EndMT or a therapeutic approach after onset of atherosclerosis.

## Discussion

EndMT is increasingly recognized as a pathological mechanism of atherosclerosis and plaque vulnerability. EndMT enables ECs to undergo a mesenchymal phenotype switch that can promote plaque growth and instability, leading to endothelial dysfunction.^7,11^ Numerous atherosclerotic risk factors - in particular inflammatory cytokines, oxidized lipids and impaired blood flow - activate EndMT via signaling pathways, mainly via TGF-β-dependent mechanisms.^6,28,30^ Conversely, certain signals such as FGF/FGFR are protective by suppressing EndMT; for example, endothelial-specific loss of FGF signaling in mouse models led to early EndMT development with ~84% increased plaque burden.^11^ In human coronary arteries, the extent of EndMT also correlates significantly with the severity of atherosclerosis.^7,11^ Taken together, this underscores the importance of endothelial plasticity in atherogenesis - but at the same time, the role of transcriptional regulation in EndMT was previously unclear.

Our results demonstrate that the transcriptional elongation machinery - specifically RNAPII elongation through the SEC - is a key regulatory layer of EndMT in atherosclerosis. Through reanalysis of human plaque scRNA-seq data using a biologically grounded three-state EndMT classification, we found *AFF4* expression significantly elevated in transitional and late mesenchymal endothelial states compared to quiescent endothelial cells.^31^ We found upregulated expression of RNA elongation complex components in EndMT-positive ECs from human plaques, indicating activation of elongation control. The analysis of over 1,000 human plaque samples from the Athero-Express biobank, using multivariate generalized linear models adjusted for clinical confounders, showed a robust association of the RNA elongation axis with plaque vulnerability features. The use of several human EndMT models confirmed this observation: in the course of EndMT, levels of RNA elongation complex components increased significantly, in parallel with the induction of mesenchymal markers. Importantly, we provide comprehensive genetic evidence supporting the central mechanistic claim: siRNA-mediated knockdown of either *AFF1* or *AFF4* in primary human ECs attenuated mesenchymal marker induction during EndMT, while *AFF4* overexpression alone was sufficient to induce mesenchymal marker expression in the absence of an EndMT stimulus. These bidirectional gain- and loss-of-function experiments establish causal sufficiency of SEC components for EndMT progression and exclude off-target activity of the small-molecule SEC inhibitors KL-1 and KL-2 as the primary explanation for the observed phenotypes. In line, we were able to show how pharmacologic inhibition of SEC attenuates EndMT both in vitro and in vivo. Specific SEC inhibitors (the peptidomimetic compounds KL-1 and KL-2^32^) led to a rapid decrease in *AFF1*/*AFF4* protein in ECs and prevented the full induction of mesenchymal genes under EndMT stimulation. Thus, SEC inhibition significantly reduced the upregulation of mesenchymal markers in the EndMT model, indicating a slowdown or attenuation of the EndMT program. At the same time, the expression of EC markers was preserved with inhibitor treatment. In a non-genetic atherosclerosis mouse model, administration of the SEC inhibitor KL-2 led to a significant reduction of plaque vulnerability, indicated by reduced plaque protrusion and a reduction of EndMT-positive cells in the plaques. Thus, our data provide consistent in vitro and in vivo results showing that transcriptional elongation via SEC represents an early, controllable step in EndMT and the development of features associated with atherosclerotic plaque vulnerability.

The mechanistic analyses support that enhanced RNAPII elongation is an early driver of the EndMT program. In unstimulated ECs, RNAPII is known to frequently reside in a promoter-proximal pause that is only resolved after an appropriate signal.^33–36^ The ChIP-seq analyses in this study showed that EndMT stimuli reduce the RNAPII pause genome-wide: Under EndMT conditions, RNAPII shifted more from promoters to the gene body, and the pausing index of many genes decreased accordingly compared to control cells. These findings indicate that EndMT-associated signals trigger the release of paused polymerases at a very early stage - a mechanism that is also used in other cellular stress or differentiation responses (e.g., heat shock or immediate-early).^34,37,38^ The SEC is central to this: AFF1/AFF4-containing SEC complex recruits P-TEFb (CDK9/cyclin T) to paused RNAPII and thus enables the transition to productive elongation.^38^ Consistent with this, we observed an accelerated rate of RNAPII elongation just minutes after induction of EndMT by 4sU-DRB pulse labeling. To evaluate the molecular hierarchy between canonical TGF-β signaling and SEC engagement, we performed reciprocal co-IP experiments in primary human ECs undergoing EndMT. AFF4 IP enriched both phosphorylated CDK9(T186) and phosphorylated SMAD2/3, while reciprocal pSMAD2/3- and pCDK9-IPs confirmed AFF4 enrichment in both directions. These tripartite interactions provide direct molecular evidence that activated SMAD2/3 — the canonical TGF-β-driven EndMT transcription factor — physically associates with the AFF4-CDK9 SEC core during EndMT, positioning SEC as an integral component of the TGF-β-driven transcriptional response rather than a parallel pathway. Genome-wide enrichment analysis of 4sU-DRB-seq elongation rates and the area under the RNAPII curve further revealed significant overrepresentation of TGF-β-related signaling pathways among SEC-regulated targets, providing convergent bioinformatic evidence for TGF-β/EMT pathway specificity.

Analysis of genome-wide 4sU-seq demonstrated that hundreds of genes exhibited increased transcription rates under EndMT, directly indicating SEC-mediated pause-release. For example, EndMT key molecules such as endoglin (*ENG*) showed a prolonged RNAPII “wave” through the gene body during early transcription, implying faster transcription - an effect that was nearly abolished by KL-1/KL-2.^39–41^ Several identified top targets were implicated before in TGF-β or ECM related signaling such as Endoglin, which is a TGF-β co-receptor amplifying SMAD2/3 signaling or ITGB1, which mediates EC migration and matrix interactions.^42,43^ Overall, SEC inhibition led to maintained high levels of pausing and thus slowed elongation. These results are consistent with studies showing that SEC inhibitors reduce the rate of RNAPII elongation and thus prevent the rapid induction of target genes.^32,44^ Thus, our data suggest that SEC-dependent RNAPII elongation is a key mechanistic step that enables early endothelial transcriptome switching during EndMT, leading to endothelial dysfunction.

The discovery of SEC-dependent elongation control as a regulator of EndMT opens up new mechanistic perspectives on plaque vulnerability. Previous approaches have mainly targeted traditional risk factors (LDL lowering, anti-inflammation), but a direct influence on endothelial plasticity has hardly been investigated.^45,46^ The results demonstrate that pharmacological and genetic blockade of transcriptional elongation can maintain ECs in a more stable endothelial phenotype despite prevailing proatherogenic stimuli. In a fully human cardiac organoid model integrating IPS-derived cardiomyocytes, primary endothelial cells, and cardiac fibroblasts, both pharmacological SEC inhibition (KL-1, KL-2) and siRNA-mediated knockdown of endoglin (the top SEC-regulated EndMT target gene) reversed two independent functional consequences of EndMT: increased fibrillar collagen deposition and reduced cardiomyocyte beating rate. To our knowledge, this represents the first demonstration that endothelial EndMT compromises cardiomyocyte function in a non-cell-autonomous manner, and that this organ-level dysfunction can be reversed by modulation of the elongation machinery. We further created a gel contractility assay for ECs undergoing EndMT, showing that both pharmacological SEC inhibition (KL-1, KL-2) and siRNA-mediated knockdown of endoglin prevented gel contractility as a feature of functional fibroblasts. In our mouse experiments, SEC inhibition reduced plaque burden and EndMT-positive cell numbers in both a prevention model and, importantly, in a therapeutic intervention model in which KL-2 was initiated after plaque establishment. The latter cohort suggests that SEC inhibition is efficacious not only prophylactically but also when applied in the clinically more relevant scenario of established atherosclerotic disease. Importantly, peripheral blood mononuclear cell transcriptome analysis and plasma cytokine ELISA (IL-1β, TNF, IL-6, IFN-γ) revealed no significant systemic anti-inflammatory or immunosuppressive effect of KL-2 administration. Combined with the absence of global transcriptional suppression in liver, heart, or blood immune cells (Supplementary Fig. 4n-p), these data argue against systemic off-target effects and support a vascular-specific mechanism. The reduction in plaque CD68^+^ macrophage content under SEC inhibition is therefore most plausibly explained as secondary to attenuated local endothelial activation and impaired vascular barrier function rather than direct effects on circulating monocytes. We have shown previously that EndMT reduces vascular barrier function resulting from impaired vascular endothelial cadherin (VE-cadherin) mediated cell-cell interaction (see Fig. 1o).^28^ These results are consistent with clinical observations that pronounced EndMT is associated with advanced, unstable atherosclerosis.^7,11^ A therapy that prevents EndMT could therefore slow the progression of atherosclerosis and prevent complications such as plaque rupture. Interestingly, some studies already indicate that modulation of the EndMT process is therapeutically possible: for example, the *HDAC3* inhibitor RGFP966 reduced EndMT and thus atherosclerosis formation in the aortic arch in the mouse model.^47,48^ In addition to their lipid-lowering effect, established drugs such as statins also appear to have EndMT-inhibiting effects - simvastatin, for example, promotes an endothelial phenotype via *KLF4*/*miR-483* and attenuation of TGF-β signaling.^49^ In this context, SEC inhibition represents a mechanistically distinct experimental approach acting at the level of transcription elongation control. SEC inhibitors have shown efficacy in oncology contexts (e.g., *MYC*-driven tumor models), supporting in vivo druggability in principle.^32^ However, translation to cardiovascular therapy will potentially require endothelial-targeted delivery strategies, careful long-term safety profiling, and adequately powered intervention studies before clinical evaluation can be considered.

Despite the promising results of the present study, some limitations should be considered. First, our in vivo data do not formally establish an endothelial cell-autonomous mechanism. While cell-type-resolved scRNA-seq analysis showed predominant SEC component upregulation in plaque endothelium, and PBMC and plasma cytokine analyses argued against systemic immunomodulation as the primary effect, the use of systemic KL-2 administration cannot exclude contributions from non-endothelial cell types. Generation of endothelial-specific *Cdh5*-Cre-driven *AFF4* conditional knockout mice represents the priority follow-up experiment to formally test endothelial cell-autonomous causality. Similarly, while in vivo EndMT was inferred from CD31/SM22, CD31/Endoglin, and N-cadherin co-staining following established approaches,^7,50^ endothelial lineage tracing using *Cdh5*-CreERT2 × Rosa26-reporter mice would provide proof of in vivo lineage conversion and is outlined as a key future experiment. Second, the long-term effect and safety of SEC inhibition is only partially known. The present in vivo intervention extended over several weeks in the mouse model; whether a permanent inhibition of transcription elongation - a fundamental cellular process - is tolerated without side effects needs to be investigated in detail.^32^ Encouragingly, our analyses showed no global transcriptional suppression in liver, heart, or peripheral blood immune cells, and no systemic increases in inflammatory cytokines. Nevertheless, possible side effects by influencing gene expression in healthy tissue remain an important aspect prior to clinical translation.^27^ Third, the extent to which subsequent therapy can reverse established EndMT remains an open question.^8^ Our therapeutic intervention cohort, in which KL-2 was administered after established hyperlipidaemia-driven plaque, demonstrated significant plaque reduction, even after onset of atherosclerosis. In the gel contraction assay, KL-2 treatment reduced contraction below the vehicle baseline, suggesting suppression of ongoing mesenchymal contractile programmes — although this does not constitute formal proof of cellular reversal. Whether ECs that have already completed the transition can be reprogrammed back to a fully functional endothelium by inhibiting SEC requires further research - so far, effective pharmacological strategies to actually revert EndMT in vivo are lacking.^51^ Fourth, we have provided functional validation of endoglin as one causal downstream effector through siRNA-mediated knockdown experiments in cardiac organoids. However, of the approximately 900 genes whose elongation is altered during EndMT, the relative contribution of additional individual targets to the integrated EndMT phenotype requires systematic investigation. Here, targeted analyses (e.g., using single-cell sequencing or functional gene silencing) could reveal further therapeutic target genes.^51,52^ Our reciprocal co-IP data demonstrate that SEC physically associates with activated SMAD2/3, suggesting that SEC inhibition might act synergistically with TGF-β pathway antagonists, a hypothesis that warrants direct testing. Fifth, all in vivo experiments were performed in male C57BL/6 mice using the *Pcsk9*-AAV8/HFD model. Sex as a biological variable, female cohorts, and additional atherosclerosis models (*ApoE*^-/-^, *Ldlr*^-/-^) represent priority extensions of the present work. The *Pcsk9*-D377Y-AAV8/HFD model was chosen because it raises LDL-cholesterol through the same pathway targeted by *Ldlr*^-/-^ mice, reduced hepatic LDL clearance, and thereby functionally phenocopies the *Ldlr*-deficient state, while avoiding both the constitutive germline knockout (and potential developmental compensation) of *Ldlr*-/- mice and the efferocytosis and T-cell-regulatory defects intrinsic to the *ApoE*^-/-^ model. It operates on an unmodified C57BL/6 background, allowing the vascular phenotype to be attributed to hypercholesterolemia rather than to a germline metabolic-gene deletion. Confirmation of the SEC-EndMT axis in genetic *Ldlr*^-/-^ and *ApoE*^-/-^ models nonetheless remains an important extension. However, the Athero-Express analyses include both sexes and were adjusted for sex in the multivariate models. Sixth, the present analysis characterizes plaque burden, plaque protrusion, CD68+ macrophage content, and in-plaque EndMT marker expression. Formal histological assessment of canonical plaque-composition features, including fibrous cap thickness, necrotic core size, collagen content, and intraplaque hemorrhage, would further strengthen the plaque vulnerability characterization. Finally, the scRNA-seq discovery cohort comprised three patients per group with proximal adjacent tissue as the within-individual control. While this design is a deliberate analytical strength, controlling for inter-individual variation and resolving rare transitional cell populations that would be invisible in bulk sequencing, formal pseudobulk inference at the donor level remains underpowered at this sample size, and we have presented the scRNA-seq findings as exploratory, complemented by the Athero-Express bulk RNA cohort (*n* = 1048). In summary, our study demonstrates a novel link between transcription kinetics and vascular pathology. The results provide proof-of-concept that modulation of RNAPII elongation can preserve the endothelial phenotype and reduce features of plaque vulnerability in experimental models. Further studies - in particular endothelial-specific genetic perturbation, lineage tracing and investigations on the safety, reversibility and optimal timing of SEC inhibition - are now needed to consider this experimental concept for translational development.

## Materials and Methods

### RNA elongation inhibitor treatment in atherosclerotic mouse model

Animal experiments were approved by the local ethics committee (approval no. TVV10/23) and followed the guidelines of the European Parliament Directive 2010/63/EU on the protection of animals used for scientific purposes. C57BL/6NTac (Charles River) male mice were maintained in a room under pathogen-free conditions with controlled 21 ± 1°C on a 12:12 h light/dark cycle (6 a.m./6 p.m.). Two different treatment models were analyzed: preventive and therapeutic treatment with KL-2. In both models, mice were first randomly divided into two groups: hypercholesterolemia-induced atherosclerosis and a control group. At 8 weeks of age, the first group received a vector containing a *Pcsk9*-D377Y gain-of-function variant (liver-specific promoter, AAV8), which was injected into the tail vein of mice, which were subsequently fed ad libitum with a modified, cholesterol (Chol)-enriched semisynthetic Clinton/Cybulsky diet (Sniff, Soest, Germany) starting at the age of 9 weeks. In the preventive model, mice of the hypercholesterolemia-induced atherosclerosis group were randomly divided into two subgroups; one group received an intraperitoneal injection of 10 mg/kg BW KL-2 in 5% DMSO in corn oil every 14 days, and the other control group received the respective volume of corn oil containing 5% DMSO for a total treatment period of 16 weeks (Endpoint: 20 weeks HFD). For the therapeutic model, mice of hypercholesterolemia-induced atherosclerosis group were randomly divided into two subgroups; one group received an intraperitoneal injection of 10 mg/kg BW KL-2 in 5% DMSO in corn oil every 7 days, and the other group received the respective volume of corn oil containing 5% DMSO for a total treatment period of 8 weeks (Endpoint: 24 weeks HFD). To validate the success of the Pcsk9-AAV8 injection, serological cholesterol levels were quantified using the LabAssay (TM) Cholesterol assay (635-50981, FUJIFILM Wako Shibayagi) and livers were analyzed using RNA sequencing.

### *En face* Oil Red O-staining of mouse aortae

For *en face* Oil Red O-staining, mouse aortae were fixed in 4% PFA overnight. Washed twice with deionized water and once with 60% isopropanol for 5 min each. Oil Red O working solution (Merck, O1391) was diluted 3:2 with deionized water and filtered. Aortae were stained in Oil Red O working solution on a rotator for 30 min at RT. Stained aortae were washed twice with 60% isopropanol and thrice with deionized water for 5 min each. After staining, aortae were opened longitudinally, and bright-field images were imaged with a Leica S9D stereomicroscope. Investigators performing histological quantification were blinded to group allocation until analysis completion.

### Immunohistochemistry and immunofluorescence of aortic root sections

Cryosections (10 µm) of mouse aortic roots were subjected to H&E stain to assess the area and protrusion of atherosclerotic plaques. Protrusion was defined as previously described.^28^ Macrophage infiltration of atherosclerotic plaques was quantified using CD68 (ab955, abcam) staining. Signal was detected using DAB substrate (550880, BD). H&E and CD68 staining were visualized with a Keyence fluorescence microscope (BZ-X) and analyzed using ImageJ software. Immunofluorescent stainings of cryosections were performed as previously described.^28^ Primary antibodies (a-CD31, AF3628, R&D systems, 1:100; a-SM22, ab14106, abcam, 1:100, both in permeabilization buffer and a-N-Cadherin, ab18203, abcam, 1:100, a-TGF-β1, ab215715, abcam, 1:100 and a-TGF-β2, 19999-1-AP, Proteintech, 1:100 all in PBS + 1% Donkey Serum (SBA-0030-01, Biozol)) were incubated overnight at 4°C. Slides were subsequently washed and incubated with secondary antibodies (a-goat-cy3, ab6949, abcam and a-rabbit-488, A-21206, Thermo Fisher), both at 1:500 in PBS, as well as Hoechst 1:200 for 1 hour at RT. Slides were counterstained with Griffonia Simplicifolia Lectin I (GSL I) isolectin B4, Biotinylated (VEC-B-1205, Biozol, 1:25 in PBS + 1% donkey serum) overnight, washed, and incubated with secondary a-Streptavidin-647 (S32357, Invitrogen, 1:500 in PBS). IF stainings for Cd31, Tgfb1, Tgfb2, Eng and Cdh2 were quantified using Leica LAS X and ImageJ. Investigators performing histological quantification were blinded to group allocation until analysis completion.

### Sirius red staining of organoids

Cryosections (10 µm) of organoids were prepared and subjected to Sirius Red staining. Sections were fixed in ice-cold methanol/acetone (1:1) and air-dried. Slides were washed once in PBS, followed by an additional wash in PBS and a brief rinse in distilled water. Sections were then incubated in Sirius Red solution (0.5 g Direct Red 80 (265548-5 G, Sigma Aldrich, US) in 500 mL picric acid (P6744-1GA, Sigma Aldrich, US)) for 1 hour at RT. After staining, slides were briefly rinsed in acidified water (0.5% acetic acid) until the washing solution appeared clear. Sections were subsequently dehydrated in 90% ethanol, followed by two washes in 100% ethanol, and one incubation in xylene (9713.3, Roth, Germany). Finally, slides were mounted using Roti®-Histokit (6638.1, Roth, Germany) mounting medium.

### ELISA

Mouse plasma was diluted 1:4 and mouse endoglin expression was quantified using the Mouse Endoglin/CD105 Quantikine ELISA Kit (MNDG00, R&D Systems) according to the manufacturer’s instructions.

### Quantitative real-time polymerase chain reaction (qPCR)

RNA was extracted using the miRNeasy Mini Kit (217004, Qiagen, DE) including on-column DNase digestion using the RNase-free DNase set (79254, Qiagen, DE). RNA from mouse liver tissue was homogenized with BD Microlances (22 G and 18 G, 304100, BD), and tissue from mouse hearts was homogenized using the Precellys Lysing Kit (P000918-LYSKO-A, Bertin Instruments) and the homogenizer Precellys Evolution Speed (Bertin Instruments) at 8,500 rpm with 3 cycles of 10 seconds and a 30 second break between cycles. RNA from homogenized mouse tissues was extracted using the miRNeasy mini Kit as described above. Eluted RNA was measured using the Denovix ds-11 photospectrometer, and 1 µg of total RNA was reverse transcribed using M-MLV (28025013, ThermoFisher Scientific, US). Transcribed cDNA was diluted to a concentration of 5 ng/µL and qPCR was performed using the QuantStudio 5 Real-Time PCR system (ThermoFisher Scientific, US). QPCR reactions were performed with PowerUp SYBR Green master mix (A25742, ThermoFisher Scientific, US) according to manufacturer’s instructions. Relative gene expression was calculated with the 2^-∆Ct^ method using *RPLP0* (human) and B2m (mouse) as reference genes. Primer sequences used in this study are listed in Supplementary table 1.

### Immunoblotting

Cells were lysed in RIPA buffer (20-188, Merck, DE + 1X HALT protease/phosphatase inhibitor cocktail (78429, ThermoFisher Scientific, US)). Total protein concentrations were determined using the Pierce BCA Protein Assay Kit (23227, ThermoFisher Scientific, US). Unless stated otherwise, 25 µg of total protein was loaded onto an SDS-Polyacrylamide gel and PAGE run using the Mini-Protean System (Bio-Rad, US). Proteins were semi-dry blotted onto a 0.45 µm nitrocellulose membrane. The membrane was subsequently blocked in 5% non-fat dry milk (NFDM) in TBS-T) for 1 hour at RT and incubated with primary antibodies overnight at 4°C. The following day, proteins were incubated with secondary antibodies conjugated to horseradish peroxidase and the chemiluminescence reaction performed using the Amersham ECL Prime Western Blotting Detection Reagent (RPN2236, GE Healthcare, UK). The following antibodies were used for immunoblotting: rabbit-anti-AFF1 (NBP-1-28728, Novus Biologicals), rabbit-anti-AFF4 (PA5-103584, ThermoFisher Scientific, US), rabbit-anti-calponin 1 (ab46794, abcam, US), rabbit-anti-VE-Cadherin (2500S, Cell signaling, US), mouse-anti-ß-tubulin (T8328, Merck, DE), rabbit-anti-calnexin (ab75801, abcam, US), goat-anti-mouse-HRP (P0447, Dako, US) and goat-anti-rabbit-HRP (P0448, Dako, US). Chemiluminescent signal was detected using the iBright imaging system (Invitrogen, US) and densitometric analysis was performed with ImageJ.

### Immunofluorescence

Chamber slides (Nunc Lab-Tek II Chamber Slide System, 154453, ThermoFisher Scientific, US) were inserted in a 12-well plate and coated with 10 µg/ mL Fibronectin (PHE0023, ThermoScientific, US) for 1 hour at 37°C. ECs were seeded at a density of 1.53 * 10 ^4^ cells per well. The following day, EndMT was initiated as described below. After 96 hours, cells were fixed with 4% PFA for 15 minutes at room temperature (RT) and subsequently permeabilized with 0.1% Triton-X-100 (T8787, Merck, DE) for 10 minutes at RT. Cells were blocked with 10% Donkey Serum (D9663, Merck, DE) for 1 hour at RT and then incubated with primary antibodies overnight at 4°C. The next day, cells were incubated with fluorescence-conjugated secondary antibodies and Hoechst 33342 DNA stain (639, Immunochemistry Technologies, US). The following antibodies were used for mesenchymal and endothelial marker detection: rabbit-anti-VE-Cadherin (1:200,2500S, Cell Signalling, US), goat-anti-TAGLN (1:100, ab10135, Abcam, US), donkey-anti-goat-555 (1:200, ab150130, Abcam, US) and donkey-anti-rabbit-488 (1:200, A-21206, ThermoFisher Scientific, US). Chamber slides were mounted with Anti-Fade DAKO Mounting Medium (S3023, DAKO, US) and imaged with the Zeiss Elyra 7 with Lattice Sim (Zeiss, DE).

### Cell Culture

Primary ECs isolated from pooled donors (CC-2519, Lonza, CH) and from single donors (C-12221, Promocell, DE) were cultivated in either EGM (CC-3124, Lonza, CH), EGM+ medium (CC-5035, Lonza, CH), or EGM-2 (CC-3162, Lonza, CH) containing all supplements. Culture Medium was supplemented with 10% heat-inactivated FBS (10082147, ThermoFisher Scientific, US). Cells were cultured at 37°C, 20% O_2_, and 5% CO_2_ and split at approximately 70% confluency using 0.25% Trypsin/EDTA (25200056, ThermoFisher Scientific, US) with subsequent inactivation through culture medium.

### siRNA-mediated knockdown

One day prior to transfection, ECs were plated at a density of 4 * 10^5^ cells per 60 mm tissue culture plate. Cells were transfected with targeting siRNA or control siRNA (final siRNA concentration 60 nM) using Lipofectamine RNAiMAX in Opti-MEM reduced serum medium (31985-070, Thermo Fisher Scientific, US). After 4 hours, the medium was replaced with full growth medium (EBM + supplements), and cells were harvested after 48 hours. siRNA sequences used: SiAFF1: GAGCUUUCUCCCUUAAUCU[dT][dT]; SiAFF4: CAGAUCUAUACCAAAGCUU[dT][dT]; SiEndoglin: CAAUGAGGCGGUGGUCAAU[dT][dT]; SiScrG: UCUCUCACAACGGGCAUUU[dT][dT].

### *AFF4* overexpression

Human umbilical vein endothelial cells (HUVECs) were cultured in EBM medium (CC-3121, Lonza, US) supplemented with the Growth Medium Supplement Pack (CC-4133, Lonza, US) at 37 °C, 5% CO₂ and 20% O₂. One day prior to transfection, cells were seeded in 60 mm culture dishes. Cells were transfected with 3 µg plasmid DNA (pcDNA or *AFF4*) using polyethyleneimine (PEI) in Opti-MEM reduced serum medium (31985-070, Thermo Fisher Scientific, US). After 4 hours, the medium was replaced with full growth medium (EBM + supplements), and cells were harvested after 72 h.

### Cardiac organoid model

Human induced pluripotent stem cell-derived cardiomyocytes (iPSC-CMs), human cardiac fibroblasts (HCFs) and human umbilical vein endothelial cells (HUVECs) were used for cardiac organoid formation. In brief, droplets of iPSC-CMs and HCFs were cultured as hanging drops at 37 °C and 5% CO₂ in a humidified atmosphere to form cardiac spheroids. After 4 days, spheroids were collected and transferred into ultra-low attachment 96-well plates. Subsequently, medium was added per well, and spheroids were allowed to recover for at least 4 hours before addition of HUVECs. After 48 hours of HUVECs attachment, organoids were referred to as the cardiac organoid model (COM). Cardiac stimulation was initiated 48 hours after HUVECs addition by culturing COM in medium containing phenylephrine (PE; P6126-5G, Sigma-Aldrich, US) for 10 days with medium exchange every second day. Contractility was assessed by counting beats per minute of at least 24 COM per condition using a light microscope. Cardiac organoids were generated from established/commercially available human induced pluripotent stem cell lines. As induced pluripotent stem cells are not subject to the German Stem Cell Act, and no new human material was collected for this study, no additional approval by an ethics committee was required.

### Collagen gel contraction assay

Contractility of HCAECs was assessed using the Cell Contraction Assay Kit (CBA-201, Cell Biolabs) with minor modifications. EC were mixed with collagen I working solution (3.0 mg/mL) at a ratio of 2:8 (cell suspension:collagen) to achieve 3 × 10⁶ cells per gel. A total of 0.5 mL per well was seeded in 24-well plates and polymerized for 1 hours at 37 °C and 5% CO₂. Gels were overlaid with 1 mL treatment medium, which was replaced every 24 hours for 72 hours. Vehicle medium consisted of EBM-2 supplemented with EGM-2 SingleQuots including 10% FCS. EndMT medium contained EBM-2 with 10% FCS and growth factor–independent supplements only. Cells were treated with either vehicle (DMSO), TGF-β2 (10 ng/mL) ± KL-1 or KL-2 (5 µM). After 72 hours, gels were released from the well edges to induce contraction. Images were acquired after 30 min, and gel area was quantified using ImageJ to calculate contraction relative to baseline. In separate experiments, for siRNA-mediated knockdown, cells were transfected with siRNA targeting endoglin or control siRNA using Lipofectamine RNAiMAX 24 hours prior to gel preparation, and the assay was performed as described.

### Co-immunoprecipitation

Co-immunoprecipitation was performed using the Magna Nuclear RIP™ Immunoprecipitation Kit (17-10520, Merck) according to the manufacturer’s instructions. For each immunoprecipitation, 1 × 10⁷ HUVECs were treated with Nuclei Isolation Buffer and lysed in RIP Cross-linked Lysis Buffer. For immunoprecipitation, Protein A/G magnetic beads were coupled to rabbit anti-pCDK9 (T186) (S2549S, Cell Signaling Technology, USA), rabbit anti-pSMAD2 (S465/467)/SMAD3 (S423/425) (8828S, Cell Signaling Technology, USA), or rabbit anti-AFF4 (PA5-103584, Thermo Fisher Scientific, USA). Antibody-coupled beads were incubated with cell lysates overnight at 4 °C. Proteins were eluted from 50 µl bead suspension and separated by SDS-polyacrylamide gel electrophoresis using the Mini-PROTEAN system (Bio-Rad, USA). Proteins were then transferred onto a 0.45 µm nitrocellulose membrane by semi-dry blotting. Membranes were blocked in 5% BSA (bovine serum albumin, Fraction V in TBS-T) for 1 hour at room temperature and incubated with primary antibodies overnight at 4 °C. The following day, membranes were incubated with HRP-conjugated secondary antibody (rabbit TrueBlot, 18-8816-33, Rockland, USA), and chemiluminescence detection was performed using SuperSignal™ West Femto (34095, Thermo Fisher Scientific, USA). The following antibodies were used for primary antibodies: rabbit-anti-pCDK9(T186) (S2549S, Cell signaling, US), rabbit-anti-pSMAD2(S465/467)/SMAD3(S423/425) (8828S, Cell signaling, US) and rabbit-anti-AFF4 (PA5-103584, ThermoFisher Scientific, US).

### Small molecule SEC inhibition

SEC inhibitors KL-1 (S2973, Selleckchem, DE) and KL-2 (CS-0087876, Universal Biologicals, US)^32^ were dissolved in DMSO (A994.2, Roth, DE) to create a 15 mM stock solution. SEC inhibition in ECs was achieved through treatment with 20 µM inhibitor for 6 h or 24 hours, respectively. For SEC inhibition in ECs undergoing EndMT, cells were repeatedly treated with 5 µM KL-1 or KL-2 at 24 h or 72 hours together with differentiation medium and TGF-β2 treatment as described in the EndMT section.

### Endothelial-to-mesenchymal-transition (EndMT)

ECs were plated at a density of 4 * 10^5^ cells per 60 mm tissue culture plate. The following day, the culture medium was changed to endothelial differentiation medium, consisting of EGM without BBE, hEGF, and ascorbic acid (CC-3124, Lonza, CH), and ECs were treated with recombinant human TGF-β2 (2-hit model, 302-B2-010, R&D Systems, US) and/or Il-1β (3-hit model, H6291, Merck, DE) at a final concentration of 10 ng/ mL. After 48 hours, ECs were re-treated with 10 ng/ mL and harvested after a total of 72 hours. Mesenchymal marker expression was examined using qPCR, immunofluorescence, or immunoblotting as described above.

### Cell viability assay

ECs were seeded at a density of 4 * 10^5^ cells per 60 mm plate and treated with small molecule inhibitors as described above on the following day. The number of dead cells was determined using the Dead Cell Apoptosis Kit with Annexin V FITC and PI for flow cytometry (V13242, ThermoFisher Scientific, US). 10% DMSO was used as a control for apoptosis. The number of apoptotic cells was determined with flow cytometry using the BD FACSLyric system (BD, US).

### Chromatin-immunoprecipitation sequencing (ChIP-Seq)

For Chromatin immunoprecipitation (ChIP), ECs were treated with SEC inhibitors KL-1 or KL-2 at 20 µM or solvent control (DMSO) for 4 hours in culture medium. ECs were subsequently treated with either culture medium (Control) or EndMT conditions (see above) as well as SEC inhibitors for 2 more hours. ChIP was performed using the *SimpleChIP Plus Enzymatic Chromatin IP* Kit (9005S, Cell Signaling, US). Approximately 2 * 10^7^ ECs were used as input per condition. Chromatin shearing was performed at 30% with 10 seconds of pulses using a sonicator, followed by 30 seconds of pausing on wet ice 3 consecutive times. Successful chromatin shearing was validated using agarose gel electrophoresis. 2% input was taken prior to the IP. 4 µg of antibody was used for each IP experiment. The following antibodies were used: RNA pol II mAb (Clone 1F4B6, 61667, Active Motif, US) and IgG (MIgG, Dianova, US). ChIP-seq libraries were prepared using the Simple ChIP ChIP-seq DNA Library Prep Kit for Illumina (56795S) and Multiplex Oligos for Illumina (Dual index primers, 47538S, both Cell Signaling, US). Sequencing was performed on a NovaSeq 6000 (Illumina, US), and Fastq reads were generated using CASAVA v.1.82 (Illumina, US).

### Bioinformatic analyses of ChIP-Seq data

Raw Fastq reads were mapped against the human reference genome (hg19) using bowtie2. Duplicates were extracted using the Picard tool v3.0.0. ChIP peaks were called with MACS2 v2.2.7.1 peak caller software^53^ using broad peak enrichment analysis. BigWig files were generated using deeptools^54^ v3.5.4, and RNAPII tracks were visualized using Integrative Genome Viewer (IGV). The heatmap was generated using deeptools on the basis of the bigwig files from the generated broad peaks with a range 3 kB up- and downstream of the annotated TSS. The pausing indices were calculated using SICER and visualized with R v.4.4.0.

### Bioinformatic analyses of liver Bulk-Seq data

After demultiplexing with the bcl2fastq software (Illumina, version 2.20), the fastq files underwent trimming and adapter removal using Trim Galore. On average, 18.2 million reads (therapeutic mouse model liver bulk RNA-seq) and 27.9 million reads (preventive mouse model liver bulk RNA-seq) per sample passed quality control and were retained for downstream analysis. Read mapping was performed against the respective reference genome (mouse mm39) using Hisat2. Gene expression quantification was achieved by read counting using FeatureCounts (R package). The resulting counts were then converted to transcripts per million (TPM) for normalization in R. Differential expression analysis was performed using the R package *edgeR*. Differences in mean expression between conditions were assessed using a negative binomial exact test.

### Sequencing of newly transcribed RNA (4sU-DRB sequencing)

Two million ECs were seeded in 150 mm tissue culture plates. After 48 hours, cells were treated with either 20 µM of SEC inhibitors KL-1 or KL-2 in culture medium for 4 hours at 37 °C. Cells were then treated with differential medium including TGF-β2 (10 ng/mL) or culture medium and SEC inhibitors for 1 hour, before the transcription inhibitor 5,6-Dichlor-benzimidazol-1-β-D-ribofuranosid (DRB, D1916, Merck, DE) was added to a final concentration of 100 µM for 1 hour. DRB was washed out with PBS; subsequently, ECs were treated with TGF-β2 in culture medium or differential medium. In this step, 4-thiouridine (4sU, T4509, Merck, DE) was added to the media, and cells were harvested after 10 min. Total RNA was extracted from cells, and DRB treatment was validated using qPCR as described above. Biotin-IP of nascent RNA was performed as previously described.^55^ Briefly, RNA labelled with 4sU was biotinylated using EZ-Link™ HPDP-Biotin (21341, ThermoFisher Scientific, US) for 1.5 hours under rotation in the dark. Biotin-labelled RNA was separated from unlabeled RNA with streptavidin-coated magnetic beads (µMACS Streptavidin Kit, 130-074-101, Miltenyi Biotech, DE), eluted with 100 mM DTT (10197777001, Merck, DE) from the beads, and precipitated overnight at -20°C with ethanol and 10 µg glycogen (R0551, ThermoFisher Scientific, US) as a precipitate enhancer. RNA pellet was purified, and a library was prepared for RNA sequencing using the TruSeq stranded total RNA library kit (20020597, Illumina, US). Sequencing was performed on a NovaSeq 6000 (Illumina, US), and Fastq reads were generated using CASAVA v.1.82 (Illumina, US).

### Bioinformatic analyses of 4sU-DRB sequencing

Raw Fastq reads were trimmed with TrimGalore and mapped to the human reference genome (hg19) using bowtie2. Read density over the gene body was plotted using deeptools. BigWig files were generated using deeptools, and track plots of newly synthesized RNA were visualized in the IGV browser. Read peaks from the newly synthesized RNA were called using the peak caller software SICER v2.0 with a window of 200 and a gap of 400. Using the different positions of the called peaks in the 0- and 10-minute samples, the RNA elongation rate in kilobases per minute was calculated and visualized using ggplot. Genes with an increased RNA elongation rate in the EndMT condition were subjected to GO analysis using topGO, analyzing the following ontologies: biological process, cell compartment, and molecular function. Significant GO enrichment was determined by Fisher's test. The polymerase wave of individual genes was calculated with the R package groHMM using “polymeraseWave” .^56^ Briefly, this algorithm applies a 3-state hidden Markov model (HMM) to GRO-seq and 4sU-seq nascent RNA data, modeling three genomic states: (1) the region upstream of the TSS, (2) the actively transcribed gene body, and (3) the 3’ polyadenylation/termination region. Transition probabilities and emission parameters were estimated from the data using the Baum-Welch algorithm. The area under the polymerase wave serves as a quantitative proxy for transcriptional output and elongation efficiency. This command uses reads from two different time points; in our study, 0 and 10 minutes after DRB washout. The difference in wave area between conditions reflects changes in the rate and processivity of Pol II elongation. The number of genes passing all quality filters (*N* = 934) represents a genome-wide characterization of the transcriptional elongation landscape rather than a gene expression comparison between biological replicates. The elongation axis was defined as the set of genes with statistically significant SEC-dependent changes in RNA elongation rate during EndMT (4sU-DRB-seq), comprising both core SEC components (AFF1, AFF4, ELL, ELL2, MLLT1, EAF1) and SEC-regulated EndMT target genes.

### Analysis of EndMT clusters in single-cell sequencing data

Raw single-cell sequencing data from atherosclerotic plaque core tissue and proximal adjacent tissue biopsied from human carotid arteries (GSE159677) was analyzed with the R toolkit Seurat v5 (Satija lab).^57^ Prior to downstream analysis, removal of cells with low quality was based on standard quality control criteria, including minimum and maximum thresholds for the number of detected genes and total UMI counts, as well as removal of cells with high mitochondrial read fractions. Doublets were identified and excluded using R package DoubletFinder. Datasets were normalized and integrated by UMAP reduction at a resolution of 0.6, and cell types were assigned to clusters using reference data from the Human Primary Cell Database. Downsampling to 12,400 cells was applied exclusively for visualization purposes in UMAP plots to ensure comparability between conditions and did not affect any quantitative analyses, including the calculation of EC/EndMT proportions. EndMT states within the EC subcluster were defined using stage-specific gene signatures derived from bulk RNA-seq time course experiments of 1-Hit and 2-Hit EndMT models. Early, transitional, and late signatures were defined based on differential gene expression between conditions, using a threshold of log2 fold change > 1 and adjusted p-value < 0.01 for comparisons of 1-Hit versus full medium control and 2-Hit versus full medium control. Early genes were defined as significantly upregulated in 1-Hit but not in 2-Hit conditions, transitional genes as those significantly upregulated in both conditions, and late genes as those specific to 2-Hit conditions. To ensure biological validity, the signatures were supplemented with established EndMT marker genes. Early-stage markers comprised *BAMBI*, *GDF15*, *SKIL*, *SMAD7*, and *TNC*. Transitional markers included *TGFBR1*, *CTNNB1*, *SFN*, *NRG1*, *TWIST1*, *ZEB2*, and *FOXC2*. Late-stage markers included *TAGLN*, *FN1*, *ITGB1*, *CNN1*, *VCAN*, and *ACTA2*. Module scores were computed using AddModuleScore (Seurat v5), and each cell was assigned to the state with the highest score. Cells with uniformly low scores across all signatures were classified as EC-quiescent.

### Analysis of human plaque samples

RNA expression data were analyzed from 1,048 carotid atherosclerotic plaque segments from the Athero-Express biobank, an ongoing biobank with extensive baseline characteristics, blood samples, and carotid atherosclerotic plaque specimens (Supplementary table 2).^29^ The study was conducted in accordance with the Declaration of Helsinki. Written informed consent was obtained from all participants, and the study was approved by the medical ethics committees of the participating hospitals (University Medical Center Utrecht, Utrecht, The Netherlands, and St. Antonius Hospital, Nieuwegein, The Netherlands) with the number TME/C-01.18 / protocol 03/114. Log2-normalized RNA expression levels of selected genes were assessed for associations with multiple histological plaque features, including macrophage content, intraplaque hemorrhage, and fat content, as previously described.^58^ In addition, associations were evaluated for the presence of severe symptoms (TIA and stroke), plaque phenotype (stable vs vulnerable), and plaque vulnerability index score (PVI). Associations between gene expression and clinical symptoms, plaque phenotype, and histological features were assessed using generalized linear models, while the association with PVI was evaluated using Spearman’s rank correlation coefficient. To correct for multiple testing, Benjamini-Hochberg (BH) adjustment was applied, with an FDR < 0.05 considered significant. Investigators performing quantification were blinded to group allocation until analysis completion.

### Statistical analysis

All data were tested for normal distribution (D’Agostino–Pearson omnibus test). For normally distributed data, two groups were compared using the unpaired, two-tailed Student’s t-test, and three or more groups by one-way ANOVA with either Tukey’s post-hoc correction (all pairwise comparisons) or Dunnett’s post-hoc correction (comparisons against a common control), as indicated in the respective figure legend. For data that did not pass normality testing, two groups were compared using the Mann–Whitney U test and three or more groups using the Kruskal–Wallis test with Dunn’s post-hoc correction. Genome-wide elongation parameters measured across matched conditions on the same set of genes (Fig. 4b, e; *n* = 934 genes) were compared using the non-parametric Friedman test with Dunn’s post-hoc correction. Differential gene expression in bulk RNA-seq data was assessed in *edgeR* using a negative binomial exact test. Enrichment analyses used Fisher’s exact test (topGO gene ontology) and ranked gene set enrichment analysis (GSEA; Hallmark gene sets, Fig. 4h). For the human Athero-Express cohort, associations between gene expression and clinical symptoms, plaque phenotype and histological features were assessed using generalized linear models adjusted for age and sex, while the association with the plaque vulnerability index (PVI) was evaluated using Spearman’s rank correlation coefficient. Multiple testing was corrected using the Benjamini–Hochberg procedure, with an FDR < 0.05 considered significant. *P*-values below 0.05 were considered significant. Data are presented as mean + SEM; *n* indicates the number of biological replicates, and technical replicates were averaged within each biological replicate prior to analysis. Quantifications were performed blinded to group allocation, and representative images correspond to the animal/section at or nearest to the group median for the primary quantified outcome. Statistical analysis and data visualization were performed using GraphPad Prism 8 and R v4.4.0.

## Supplementary information

Supplementary Materials

## Data Availability

Raw single-cell sequencing data from atherosclerotic plaque core tissue and proximal adjacent tissue biopsied from human carotid arteries are available (GSE159677). Sequencing data are deposited at GSE330230.
