# Supplementary material for: Targeting super elongation complex-driven RNA polymerase II elongation reduces plaque vulnerability
Source: Signal Transduct Target Ther. 2026 Sep 17;11:385. doi: 10.1038/s41392-026-02962-4 (PMC13583046; doi:10.1038/s41392-026-02962-4)
Supplement: Supplementary file 1 [file 41392_2026_2962_MOESM1_ESM.docx]

Supplementary Materials for

Targeting super elongation complex–driven RNA polymerase II elongation reduces plaque vulnerability

Karoline E. Kokot, Sebastian Reichardt, Christian Menedo, Stephan Erbe, Jasmin M. Kneuer, Kaylin C.A. Palm, Julian Hoba, Michael Andritschke, Mirko Katzmann, Annika Reuser, Moritz von Scheidt, Michal Mokry, Junjie Xiao, Bilal N. Sheikh, Nora Klöting, Ulrich Laufs and Jes-Niels Boeckel

Correspondence to: Boeckel@medizin.uni-leipzig.de

**This PDF file includes:**

Figures. S1 to S6

Tables S1 to S3

**Figure. S1.**

(a) Summary of detected UMI counts, features and mitochondrial and ribosomal gene fractions for the scRNA-seq carotid artery dataset (Proximal adjacent vessel section (CV): n = 3, Atherosclerotic plaque section (AP): n = 3). Human carotid artery plaques and proximal adjacent vessel sections (used as control) from the same patient were analyzed. (b) Dotplot showing gene expression profiles of *AFF1* and *AFF4* in smooth muscle cells, immune cells and fibroblasts of CV and AP sections (CV: n = 3, AP: n = 3). (c) mRNA expression of common proteins forming the SEC: ELL1, ELL2, AF9, ENL and CDK9 in HUVEC, HCAEC, HCM and HCASMC (n = 2-3). (d) Heatmap depicting expression of ECM-associated genes in RNA sequencing data of HCAEC under control conditions and undergoing EndMT (1-Hit, 2-Hit) cell culture model (n = 4). (e-h) Validation of EndMT in EndMT cell culture model. mRNA expression of mesenchymal markers (e) SM22, (f) *CNN1*, (g) VCAN and of the endothelial marker (h) *LYVE1* in HUVECs and HUVECs undergoing EndMT (2-Hit model) was determined by qPCR (n = 4, e/f/g: t-test; h: Mann-Whitney test). (i) *AFF1* mRNA expression and (j) *AFF4* mRNA expression in EndMT samples compared to controls (n = 3, t-test). (k) Representative immunoblot depicting VE-Cadherin and Calponin protein levels under control and EndMT conditions. Quantification of (l) VE-Cadherin and (m) Calponin protein expression in HUVECs undergoing EndMT determined by immunoblotting (n=5, l: Mann-Whitney test, m: t-test). Quantification of (n) VE-Cadherin and (o) *AFF1* protein level in HUVECs undergoing EndMT (2-Hit, 3-Hit) determined by immunoblotting normed to β-actin (n=5).

Figure. S2.

Annexin V-positive (+) HUVECs after treatment with increasing concentrations of KL-1 and KL-2 for 24 h determined by flow cytometry. 10% DMSO serves as a positive control (n = 3).

Figure. S3.

*COL8A1* calculated using the Hidden Markov model with groHMM depicting the read changes between 10 and 0 minutes after EndMT induction with or without KL-1 and KL-2 inhibition.

Figure. S4.

**(a-c)** mRNA expression of inflammation markers **(a)** *Il1b*, **(b)** *Tnf* and **(c)** *Il6* in mice PBMC treated with *Pcsk9*-AAV8 (n = 7 (DMSO) vs. n = 8 (KL-2) or PBS after 20 weeks as analyzed by RNA seq. (Ordinary one-way ANOVA with Dunnett's multiple comparisons test). **(d-g)** Plasma protein levels of inflammation markers **(d)** Il-1β, **(e)** Tnf-α, **(f)** Il-6 and **(g)** Ifn-γ of atherosclerotic mice with or without KL-2 treatment determined by ELISA (n = 7 (DMSO) vs. n = 8 (KL-2). **(h-m)** Vascular inflammation marker **(h)** *Icam1*, **(i)** *Vcam1* and SEC target genes **(j)** *Itgb1*, **(k)** *Klf3*, **(l)** *Cd9* and **(m)** *Nexn* mRNA expression in livers of mice treated with *Pcsk9*-AAV8 (n = 4 (DMSO) vs. 3 (KL-2) or PBS after 20 weeks as analyzed by RNA seq.; One-way ANOVA with Dunnett's multiple comparisons test). Analysis of overall transcription in **(n)** peripheral blood mononuclear cells (PBMCs), **(o)** liver and **(p)** heart tissue of mice treated with *Pcsk9*-AAV8 and vehicle or KL-2.

Figure. S5.

(a) Representative images and (b) quantification of EndMT-positive EC as indicated by Sm22+ Cd31+ cells in atherosclerotic plaques (n = 5-8, t-test). All bar graphs represent mean + SEM, * indicates P < 0.05.

Figure. S6.

(a) Representative depiction of immunofluorescence staining of N-Cadherin (*Cdh2*) and Isolectin B4 in frozen aortic root sections and (b) quantification of Cdh2 positive cells in atherosclerotic plaques (n = 5-10, One way ANOVA, Dunnett’s multiple comparisons test). (c) Representative depiction of immunofluorescence staining of Tgf-β1 and Isolectin B4 in frozen aortic root sections and (d) quantification of Tgf-β1 positive cells in atherosclerotic plaques (n = 5-10, Kruskal-Wallis test with Dunn’s multiple comparisons test). (e) Representative depiction of immunofluorescence staining of Tgf-β2 and Isolectin B4 in frozen aortic root sections and (f) quantification of Tgf-β2 positive cells in atherosclerotic plaques (n = 5-10, One way ANOVA, Dunnett’s multiple comparisons test). All bar graphs represent mean + SEM, * indicates P < 0.05.

Table S1. Primer sequences

| Name | Sequence (F+R) 5->3 | Species |
| --- | --- | --- |
| *RPLP0* | TCGACAATGGCAGCATCTAC | Human |
|  | ATCCGTCTCCACAGACAAGG |  |
| *CDH5* | ACGCCTCTGTCATGTACCAA | Human |
|  | ACGATCTCATACCTGGCCTG |  |
| *VWF* | GAGTACGCCCGGACCTGT | Human |
|  | TTCATTGATGTGCAGGCTCT |  |
| *AFF1* | GCCAAGCTCACCAAACTGAAG | Human |
|  | AAAGGAGGCGGCCATGAATG |  |
| *AFF2* | CAAAACCCAAGCGTCAGACA | Human |
|  | GGAGGGAGCAAGCAGATAGT |  |
| *AFF3* | GGCCACCTCCACCAGTAATA | Human |
|  | GATGCATCAGAGAGGGGTGA |  |
| *AFF4* | GAAAAGCACACGAGAGAGGC | Human |
|  | GGTTTCTTGCTCTCACTGGC |  |
| *ELL1* | GACAAGGACGCGCTGGAT | Human |
|  | AGTCCTTCTGCACATCCTTG |  |
| *ELL2* | CGAGAAAGAATGACCCAGGC | Human |
|  | GCTTGAGGTGCTTTCCGAAT |  |
| *AF9* | CCCAATGATTCAGATGTGGAGG | Human |
|  | TGGTCTGGGATGGTGTGAAG |  |
| *ENL* | GAGTTCCGGTACAAGCTCCT | Human |
|  | TGGTTTGGTCTTCTTGGGGT |  |
| *CDK9* | GTGTTCAAGGCCAGGCAC | Human |
|  | GCAAGGCTGTAATGGGGAA |  |
| *SM22* | AAGAATGATGGGCACTACCG | Human |
|  | ATGACATGCTTTCCCTCCTG |  |
| *CNN1* | CTGGCTGCAGCTTATTGATG | Human |
|  | CTGAGAGAGTGGATCGAGGG |  |
| *LYVE1* | TTCCAGTGAGCCGACAGTTT | Human |
|  | CTGGAATGCACGAGTTAGTCC |  |
| *VCAN* | ATGGATTCCGTTCTCCCCAG | Human |
|  | CACTCTTTTGCAGCCTCCTC |  |
| *B2m* | TTCTGGTGCTTGTCTCACTGA | Mouse |
|  | CAGTATGTTCGGCTTCCCATTC |  |
| *Eng* | TGACGTTTACCACCAGCCA | Mouse |
|  | GTCTCCGTGCCATTTTGCTT |  |

Table S2. Baseline patient data of the Athero-Express cohort

| **Baseline Table Athero-Express RNA Cohort** | | | |
| --- | --- | --- | --- |
|  | less vulnerable | more vulnerable | p-value |
| **n = 1,048** | 703 | 345 |  |
| **Sex = male (%)** | 509 (72.4) | 254 (73.6) | 0.732 |
| **Age (median [IQR])** | 70.00 [63.00, 75.00] | 70.00 [62.00, 76.00] | 0.963 |
| **Diabetes = yes (%)** | 160 (23.3) | 61 (17.9) | 0.061 |
| **Smoking status = yes (%)** | 239 (34.1) | 118 (34.6) | 0.938 |
| **Kidney function (%)** |  |  | 0.825 |
| **Normal kidney function** | 126 (18.4) | 70 (20.7) |  |
| **CKD 2 (Mild)** | 385 (56.1) | 185 (54.7) |  |
| **CKD 3 (Moderate)** | 163 (23.8) | 79 (23.4) |  |
| **CKD 4 (Severe)** | 11 (1.6) | 4 (1.2) |  |
| **CKD 5 (Failure)** | 1 (0.1) | 0 (0.0) |  |
| **Statin use = yes (%)** | 542 (77.2) | 282 (81.7) | 0.109 |
| **LDL cholesterol levels (mg/dL)**  **(median [IQR])** | 2.70 [2.00, 3.34] | 2.60 [2.00, 3.40] | 0.764 |
| **HDL cholesterol levels (mmol/L)**  **(median [IQR])** | 1.11 [0.91, 1.39] | 1.10 [0.89, 1.34] | 0.354 |
| **Total cholesterol levels (mg/dL)**  **(median [IQR])** | 4.64 [3.80, 5.46] | 4.54 [3.76, 5.51] | 0.803 |
| **Triglyceride levels (mmol/L)**  **(median [IQR])** | 1.31 [0.99, 1.81] | 1.41 [1.06, 2.10] | 0.045 |
| **Diastolic (median [IQR])** | 80.00 [70.25, 90.00] | 81.00 [70.75, 90.00] | 0.298 |
| **Systolic (median [IQR])** | 150.00 [135.00, 169.00] | 152.00 [135.00, 170.00] | 0.302 |
| **eGFR (mL/min/1.73 m²)**  **(mean (SD))** | 72.72 (20.16) | 73.49 (20.90) | 0.572 |
| **BMI (kg/m²) (median [IQR])** | 26.20 [24.09, 28.73] | 26.09 [24.15, 28.31] | 0.526 |
| **Plasma hsCRP (median [IQR])** | 1.92 [0.92, 4.47] | 1.75 [0.70, 4.31] | 0.339 |
| **CAD history = yes (%)** | 237 (33.8) | 99 (28.7) | 0.114 |
| **Major events (endpoints) (%)** | 86 (12.4) | 45 (13.1) | 0.816 |
| **Symptomatic (%)** | 438 (65.8) | 248 (77.5) | <0.001 |
| **Plaque macrophage content = moderate/heavy (%)** | 339 (52.2) | 201 (66.3) | <0.001 |
| **Plaque SMC content =**  **moderate/heavy (%)** | 431 (66.2) | 204 (66.9) | 0.894 |
| **Plaque fat content = >10% (%)** | 466 (69.8) | 266 (83.9) | <0.001 |
| **Plaque collagen content =**  **moderate/heavy (%)** | 492 (79.7) | 226 (78.7) | 0.798 |

CKD, chronic kidney disease; HDL, high-density lipoprotein; IQR, interquartile range; BMI, body mass index; CAD, coronary artery disease; SMC, smooth muscle cell

Table S3. Statistical tests

| **Figure** | **Panel** | **Biological n** | **Independent replicate definition** | **Quantification unit** | **statistical test used** | **exact p-value** |
| --- | --- | --- | --- | --- | --- | --- |
| **1** | **m/n/p/q** | Control: 4 | independently treated cell cultures | one bulk RNA-seq sample per biological replicate | **negative binomial exact test** | p<0.05 |
|  |  | EndMT (1-Hit): 4 |  |  |  |  |
|  |  | EndMT (2-Hit): 4 |  |  |  |  |
| **1** | **r** | Control: 4 | independently treated cell cultures | one bulk RNA-seq sample per biological replicate | **One-way ANOVA with Dunnett's multiple comparisons test, with a single pooled variance** | Control vs. 1-Hit: 0.0062 |
|  |  | EndMT, 1-Hit: 4 |  |  |  | Control vs. 2-Hit: 0.0136 |
|  |  | EndMT, 2-Hit: 4 |  |  |  |  |
| **1** | **s** | Control: 4 | independently treated cell cultures | one bulk RNA-seq sample per biological replicate | **One-way ANOVA with Dunnett's multiple comparisons test, with a single pooled variance** | Control vs. 1-Hit: 0.0221 |
|  |  | EndMT, 1-Hit: 4 |  |  |  | Control vs. 2-Hit: 0.0244 |
|  |  | EndMT, 2-Hit: 4 |  |  |  |  |
| **1** | **v** | Control: 5 | independently treated cell cultures | one immunoblot sample per biological replicate | **One-way ANOVA with Dunnett's multiple comparisons test, with a single pooled variance** | Control vs. 2-Hit: 0.0324 |
|  |  | EndMT, 2-Hit: 5 |  |  |  | Control vs. 3-Hit: 0.0075 |
|  |  | EndMT, 3-Hit: 5 |  |  |  |  |
| **1** | **w** | Control: 5 | independently treated cell cultures | one immunoblot sample per biological replicate | **One-way ANOVA with Dunnett's multiple comparisons test, with a single pooled variance** | Control vs. 2-Hit: 0.0375 |
|  |  | EndMT, 2-Hit: 5 |  |  |  |  |
|  |  | EndMT, 3-Hit: 5 |  |  |  |  |
|  |  |  |  |  |  |  |
| **Figure** | **Panel** | **Biological n** | **Independent replicate definition** | **Quantification unit** | **statistical test used** | **exact p-value** |
| **2** | **b** | Control: 4 | independently treated cell cultures | one immunoblot sample and image per biological replicate | **One-way ANOVA with Tukey's multiple comparisons test, with a single pooled variance** | DMSO vs. KL-1: 0.0424 |
|  |  | KL-1: 4 |  |  |  | DMSO vs. KL-2: 0.0096 |
|  |  | KL-2: 4 |  |  |  |  |
| **2** | **d** | Control: 4 | independently treated cell cultures | one immunoblot sample and image per biological replicate | **One-way ANOVA with Tukey's multiple comparisons test, with a single pooled variance** | Control vs. KL-1: 0.0460 |
|  |  | KL-1: 4 |  |  |  | Control vs. KL-2: 0.0138 |
|  |  | KL-2: 4 |  |  |  |  |
| **2** | **f** | DMSO: 3 | independently treated cell cultures | one flow cytometry sample per biological replicate | **One-way ANOVA with Tukey's multiple comparisons test, with a single pooled variance** | All vs. DMSO (10%): < 0.001 |
|  |  | KL-1 (20 µM): 3 |  |  |  |  |
|  |  | KL-1 (20 µM): 3 |  |  |  |  |
|  |  | KL-1 (20 µM): 3 |  |  |  |  |
|  |  | KL-2 (20 µM): 3 |  |  |  |  |
|  |  | KL-2 (20 µM): 3 |  |  |  |  |
|  |  | KL-2 (20 µM): 3 |  |  |  |  |
|  |  | DMSO (10%): 3 |  |  |  |  |
| **2** | **g** | DMSO: 3 | independently treated cell cultures | one cDNA sample per biological replicate | **One-way ANOVA with Tukey's multiple comparisons test, with a single pooled variance** | DMSO vs. EndMT + DMSO: 0.0176 |
|  |  | EndMT + DMSO: 3 |  |  |  | EndMT + DMSO vs. EndMT + KL-1 (5 µM): 0.057 |
|  |  | EndMT + KL-1 (2.5 µM): 3 |  |  |  | EndMT + DMSO vs. EndMT + KL-1 (10 µM): 0.039 |
|  |  | EndMT + KL-1 (5 µM): 3 |  |  |  | EndMT + DMSO vs. EndMT + KL-2 (5 µM): 0.0097 |
|  |  | EndMT + KL-1 (10 µM): 3 |  |  |  | EndMT + DMSO vs. EndMT + KL-1 (10 µM): 0.0045 |
|  |  | EndMT + KL-2 (2.5 µM): 3 |  |  |  |  |
|  |  | EndMT + KL-2 (5 µM): 3 |  |  |  |  |
|  |  | EndMT + KL-2 (10 µM): 3 |  |  |  |  |
| **2** | **h** | DMSO: 4 | independently treated cell cultures | one cDNA sample per biological replicate | **One-way ANOVA with Tukey's multiple comparisons test, with a single pooled variance** | DMSO vs. EndMT + DMSO: 0.0293 |
|  |  | EndMT + DMSO: 4 |  |  |  | EndMT + DMSO vs. EndMT + KL-1: 0.0391 |
|  |  | EndMT + ActD (1:1000): 4 |  |  |  | EndMT + DMSO vs. EndMT + KL-2: 0.0473 |
|  |  | EndMT + DRB (1:1000): 4 |  |  |  |  |
|  |  | EndMT + KL-1: 4 |  |  |  |  |
|  |  | EndMT + KL-2: 3 |  |  |  |  |
| **2** | **i** | EndMT + DMSO: 3 | independently treated cell cultures | one cDNA sample per biological replicate | **One-way ANOVA with Dunnett's multiple comparisons test, with a single pooled variance** | EndMT + DMSO vs. EndMT + KL-1: 0.0005 |
|  |  | EndMT + KL-1: 3 |  |  |  | EndMT + DMSO vs. EndMT + KL-2: 0.0005 |
|  |  | EndMT + KL-2: 3 |  |  |  |  |
| **2** | **j** | EndMT + DMSO: 3 | independently treated cell cultures | one cDNA sample per biological replicate | **One-way ANOVA with Dunnett's multiple comparisons test, with a single pooled variance** | EndMT + DMSO vs. EndMT + KL-1: 0.0098 |
|  |  | EndMT + KL-1: 3 |  |  |  | EndMT + DMSO vs. EndMT + KL-2: 0.0089 |
|  |  | EndMT + KL-2: 3 |  |  |  |  |
| **2** | **k** | EndMT + DMSO: 3 | independently treated cell cultures | one cDNA sample per biological replicate | **Kruskal-Wallis test with Dunn's multiple comparisons test** | ns |
|  |  | EndMT + KL-1: 3 |  |  |  |  |
|  |  | EndMT + KL-2: 3 |  |  |  |  |
| **2** | **m** | Control: 5 | independently treated cell cultures | one immunoblot sample and image per biological replicate | **One-way ANOVA with Tukey's multiple comparisons test, with a single pooled variance** | Control vs. EndMT, 2-Hit: <0.0001 |
|  |  | EndMT, 2-Hit: 5 |  |  |  | Control vs. EndMT, 3-Hit: <0.0001 |
|  |  | EndMT, 2-Hit + KL-1: 5 |  |  |  | EndMT, 2-Hit vs. EndMT, 2-Hit + KL-1: <0.0001 |
|  |  | EndMT, 2-Hit + KL-2: 5 |  |  |  | EndMT, 2-Hit vs. EndMT, 2-Hit + KL-2: <0.0001 |
|  |  | EndMT, 3-Hit: 5 |  |  |  | EndMT, 3-Hit vs. EndMT, 3-Hit + KL-1: <0.0001 |
|  |  | EndMT, 3-Hit + KL-1: 5 |  |  |  | EndMT, 3-Hit vs. EndMT, 3-Hit + KL-2: <0.0001 |
|  |  | EndMT, 3-Hit + KL-2: 5 |  |  |  |  |
| **2** | **n** | pcDNA: 6 | independently treated cell cultures | one cDNA sample per biological replicate | **t-test** | pcDNA vs. AFF4: 0.0070 |
|  |  | AFF4: 6 |  |  |  |  |
| **2** | **o** | pcDNA: 6 | independently treated cell cultures | one cDNA sample per biological replicate | **t-test** | pcDNA vs. AFF4: 0.0287 |
|  |  | AFF4: 6 |  |  |  |  |
| **2** | **p** | pcDNA: 5 | independently treated cell cultures | one cDNA sample per biological replicate | **t-test** | pcDNA vs. AFF4: 0.0239 |
|  |  | AFF4: 5 |  |  |  |  |
| **2** | **q** | EndMT + SiScrG: 4 | independently treated cell cultures | one cDNA sample per biological replicate | **t-test** | EndMT + SiScrG vs. EndMT + siAFF1: 0.0004 |
|  |  | EndMT + siAFF1: 4 |  |  |  |  |
| **2** | **r** | Control: 4 | independently treated cell cultures | one cDNA sample per biological replicate | **One-way ANOVA with Tukey's multiple comparisons test, with a single pooled variance** | Control vs. EndMT + SiScrG: < 0.0001 |
|  |  | EndMT + SiScrG: 4 |  |  |  | EndMT + SiScrG vs. EndMT + siAFF1: 0.0028 |
|  |  | EndMT + siAFF1: 4 |  |  |  |  |
| **2** | **s** | Control: 4 | independently treated cell cultures | one cDNA sample per biological replicate | **One-way ANOVA with Tukey's multiple comparisons test, with a single pooled variance** | Control vs. EndMT + SiScrG: 0.0004 |
|  |  | EndMT + SiScrG: 4 |  |  |  | EndMT + SiScrG vs. EndMT + siAFF1: 0.0342 |
|  |  | EndMT + siAFF1: 4 |  |  |  |  |
| **2** | **t** | EndMT + SiScrG: 4 | independently treated cell cultures | one cDNA sample per biological replicate | **t-test** | EndMT + SiScrG vs. EndMT + siAFF4: 0.0013 |
|  |  | EndMT + siAFF4: 4 |  |  |  |  |
| **2** | **u** | Control: 4 | independently treated cell cultures | one cDNA sample per biological replicate | **One-way ANOVA with Tukey's multiple comparisons test, with a single pooled variance** | Control vs. EndMT + SiScrG: < 0.0001 |
|  |  | EndMT + SiScrG: 4 |  |  |  | EndMT + SiScrG vs. EndMT + siAFF4: 0.0028 |
|  |  | EndMT + siAFF4: 4 |  |  |  |  |
| **2** | **v** | Control: 4 | independently treated cell cultures | one cDNA sample per biological replicate | **One-way ANOVA with Tukey's multiple comparisons test, with a single pooled variance** | Control vs. EndMT + SiScrG: 0.0005 |
|  |  | EndMT + SiScrG: 4 |  |  |  | EndMT + SiScrG vs. EndMT + siAFF4: 0.0092 |
|  |  | EndMT + siAFF4: 4 |  |  |  |  |
|  |  |  |  |  |  |  |
| **Figure** | **Panel** | **Biological n** | **Independent replicate definition** | **Quantification unit** | **statistical test used** | **exact p-value** |
| **4** | **b** | Control: 934 (genes) | independently treated cell cultures | one 4sU-seq sample per biological replicate | **Friedman test with Dunn's multiple comparisons test** | Control vs. EndMT: < 0.0001 |
|  |  | EndMT + DMSO: 934 (genes) |  |  |  | EndMT vs. EndMT + KL-1: < 0.0001 |
|  |  | EndMT + KL-1: 934 (genes) |  |  |  | EndMT vs. EndMT + KL-2: < 0.0001 |
|  |  | EndMT + KL-2: 934 (genes) |  |  |  |  |
| **4** | **e** | Control: 934 (genes) | independently treated cell cultures | one 4sU-seq sample per biological replicate | **Friedman test with Dunn's multiple comparisons test** | Control vs. EndMT: < 0.0001 |
|  |  | EndMT + DMSO: 934 (genes) |  |  |  | EndMT vs. EndMT + KL-1: < 0.0001 |
|  |  | EndMT + KL-1: 934 (genes) |  |  |  | EndMT vs. EndMT + KL-2: < 0.0001 |
|  |  | EndMT + KL-2: 934 (genes) |  |  |  |  |
| **4** | **h** | Control: 1 | independently treated cell cultures | one 4sU-seq sample per biological replicate | **Ranked-GSEA** | p<0.05 |
|  |  | EndMT + DMSO: 1 |  |  |  |  |
| **4** | **j** | IP: IgG: 4 | independently treated cell cultures | one immunoblot sample and image per biological replicate | **t-test** | IP: IgG vs. IP: pSMAD2/3: 0.0309 |
|  |  | IP: pSMAD2/3: 4 |  |  |  |  |
| **4** | **k** | IP: IgG: 4 | independently treated cell cultures | one immunoblot sample and image per biological replicate | **Mann-Whitney test** | IP: IgG vs. IP: pSMAD2/3: 0.0286 |
|  |  | IP: pSMAD2/3: 4 |  |  |  |  |
| **4** | **m** | IP: IgG: 4 | independently treated cell cultures | one immunoblot sample and image per biological replicate | **Mann-Whitney test** | IP: IgG vs. IP: pCDK9: 0.0286 |
|  |  | IP: pCDK9: 4 |  |  |  |  |
| **4** | **n** | IP: IgG: 4 | independently treated cell cultures | one immunoblot sample and image per biological replicate | **t-test** | IP: IgG vs. IP: pCDK9: 0.0146 |
|  |  | IP: pCDK9: 4 |  |  |  |  |
| **4** | **o** | IP: IgG: 4 | independently treated cell cultures | one immunoblot sample and image per biological replicate | **t-test** | IP: IgG vs. IP: pCDK9: 0.0031 |
|  |  | IP: pCDK9: 4 |  |  |  |  |
| **4** | **q** | IP: IgG: 4 | independently treated cell cultures | one immunoblot sample and image per biological replicate | **t-test** | IP: IgG vs. IP: pAFF4: 0.0151 |
|  |  | IP: AFF4: 4 |  |  |  |  |
| **4** | **r** | IP: IgG: 4 | independently treated cell cultures | one immunoblot sample and image per biological replicate | **t-test** | IP: IgG vs. IP: pAFF4: 0.0109 |
|  |  | IP: AFF4: 4 |  |  |  |  |
| **4** | **s** | IP: IgG: 4 | independently treated cell cultures | one immunoblot sample and image per biological replicate | **t-test** | IP: IgG vs. IP: pAFF4: 0.0192 |
|  |  | IP: AFF4: 4 |  |  |  |  |
|  |  |  |  |  |  |  |
| **Figure** | **Panel** | **Biological n** | **Independent replicate definition** | **Quantification unit** | **statistical test used** | **exact p-value** |
| **5** | **b** | DMSO: 29 | independently treated organoids | one count per organoid at baseline and 24 h (EndMT induction) | **One-way ANOVA with Dunnett's multiple comparisons test, with a single pooled variance** | DMSO vs. EndMT + DMSO: < 0.0001 |
|  |  | EndMT + DMSO: 26 |  |  |  | EndMT vs. EndMT + KL-1: 0.0005 |
|  |  | EndMT + KL-1: 30 |  |  |  | EndMT vs. EndMT + KL-2: < 0.0001 |
|  |  | EndMT + KL-2: 27 |  |  |  |  |
| **5** | **c** | DMSO: 29 | independently treated organoids | one count per organoid at baseline and 48 h (EndMT induction) | **One-way ANOVA with Dunnett's multiple comparisons test, with a single pooled variance** | DMSO vs. EndMT + DMSO: < 0.0001 |
|  |  | EndMT + DMSO: 26 |  |  |  | EndMT vs. EndMT + KL-1: 0.0159 |
|  |  | EndMT + KL-1: 30 |  |  |  | EndMT vs. EndMT + KL-2: < 0.0001 |
|  |  | EndMT + KL-2: 27 |  |  |  |  |
| **5** | **d** | DMSO: 29 | independently treated organoids | one count per organoid at baseline and 72 h (EndMT induction) | **One-way ANOVA with Dunnett's multiple comparisons test, with a single pooled variance** | DMSO vs. EndMT + DMSO: < 0.0001 |
|  |  | EndMT + DMSO: 26 |  |  |  | EndMT vs. EndMT + KL-1: 0.0187 |
|  |  | EndMT + KL-1: 30 |  |  |  | EndMT vs. EndMT + KL-2: < 0.0001 |
|  |  | EndMT + KL-2: 27 |  |  |  |  |
| **5** | **f** | DMSO: 5 | independently treated organoids | one organoid and image per biological replicate | **One-way ANOVA with Dunnett's multiple comparisons test, with a single pooled variance** | DMSO vs. EndMT + DMSO: 0.0004 |
|  |  | EndMT + DMSO: 5 |  |  |  | EndMT vs. EndMT + KL-1: < 0.0001 |
|  |  | EndMT + KL-1: 5 |  |  |  | EndMT vs. EndMT + KL-2: 0.0002 |
|  |  | EndMT + KL-2: 5 |  |  |  |  |
| **5** | **h** | DMSO: 4 | independently treated cells in collagen gel | one cell collagen gel and image per biological replicate | **One-way ANOVA with Dunnett's multiple comparisons test, with a single pooled variance** | DMSO vs. EndMT + DMSO: 0.0073 |
|  |  | EndMT + DMSO: 4 |  |  |  | EndMT vs. EndMT + KL-1: 0.0088 |
|  |  | EndMT + KL-1: 4 |  |  |  | EndMT vs. EndMT + KL-2: < 0.0001 |
|  |  | EndMT + KL-2: 4 |  |  |  |  |
| **5** | **i** | Control: 28 | independently treated organoids | one count per organoid at baseline and 24 h (EndMT induction) | **One-way ANOVA with Dunnett's multiple comparisons test, with a single pooled variance** | Control vs. EndMT + SiScrG: < 0.0001 |
|  |  | EndMT + SiScrG: 32 |  |  |  | EndmT + SiScrG vs. EndMT + siEndoglin: 0.0146 |
|  |  | EndMT + siEndoglin: 24 |  |  |  |  |
| **5** | **j** | Control: 28 | independently treated organoids | one count per organoid at baseline and 48 h (EndMT induction) | **One-way ANOVA with Dunnett's multiple comparisons test, with a single pooled variance** | Control vs. EndMT + SiScrG: < 0.0001 |
|  |  | EndMT + SiScrG: 32 |  |  |  | EndmT + SiScrG vs. EndMT + siEndoglin: 0.0339 |
|  |  | EndMT + siEndoglin: 24 |  |  |  |  |
| **5** | **k** | Control: 28 | independently treated organoids | one count per organoid at baseline and 72 h (EndMT induction) | **One-way ANOVA with Dunnett's multiple comparisons test, with a single pooled variance** | Control vs. EndMT + SiScrG: < 0.0001 |
|  |  | EndMT + SiScrG: 32 |  |  |  | EndmT + SiScrG vs. EndMT + siEndoglin: 0.0001 |
|  |  | EndMT + siEndoglin: 24 |  |  |  |  |
| **5** | **m** | Control: 5 | independently treated organoids | one organoid and image per biological replicate | **One-way ANOVA with Dunnett's multiple comparisons test, with a single pooled variance** | Control vs. EndMT + SiScrG: 0.0048 |
|  |  | EndMT + SiScrG: 5 |  |  |  | EndmT + SiScrG vs. EndMT + siEndoglin: 0.0325 |
|  |  | EndMT + siEndoglin: 5 |  |  |  |  |
| **5** | **o** | Control: 4 | independently treated cells in collagen gel | one cell collagen gel and image per biological replicate | **One-way ANOVA with Dunnett's multiple comparisons test, with a single pooled variance** | Control vs. EndMT + SiScrG: < 0.0001 |
|  |  | EndMT + SiScrG: 4 |  |  |  | EndmT + SiScrG vs. EndMT + siEndoglin: < 0.0001 |
|  |  | EndMT + siEndoglin: 4 |  |  |  |  |
|  |  |  |  |  |  |  |
| **Figure** | **Panel** | **Biological n** | **Independent replicate definition** | **Quantification unit** | **statistical test used** | **exact p-value** |
| **6** | **c** | Normal Chow: 6 | independently treated animals | one serum per biological replicate | **One-way ANOVA with Dunnett's multiple comparisons test, with a single pooled variance** | Normal Chow vs. Pcsk9-AAV8/HFD + DMSO: 0.0002 |
|  |  | Pcsk9-AAV8/HFD + DMSO: 7 |  |  |  | Normal Chow vs. Pcsk9-AAV8/HFD + KL-2: < 0.0001 |
|  |  | Pcsk9-AAV8/HFD + KL-2: 8 |  |  |  |  |
| **6** | **e** | Normal Chow: 7 | independently treated animals | one aorta and image per biological replicate | **One-way ANOVA with Dunnett's multiple comparisons test, with a single pooled variance** | Normal Chow vs. Pcsk9-AAV8/HFD + DMSO: 0.0011 |
|  |  | Pcsk9-AAV8/HFD + DMSO: 11 |  |  |  | Pcsk9-AAV8/HFD + DMSO vs. Pcsk9-AAV8/HFD + KL-2: 0.0030 |
|  |  | Pcsk9-AAV8/HFD + KL-2: 7 |  |  |  |  |
| **6** | **g** | Normal Chow: 8 | independently treated animals | one aortic root section and image per biological replicate | **One-way ANOVA with Dunnett's multiple comparisons test, with a single pooled variance** | Normal Chow vs. Pcsk9-AAV8/HFD + DMSO: 0.0005 |
|  |  | Pcsk9-AAV8/HFD + DMSO: 12 |  |  |  | Pcsk9-AAV8/HFD + DMSO vs. Pcsk9-AAV8/HFD + KL-2: 0.0041 |
|  |  | Pcsk9-AAV8/HFD + KL-2: 6 |  |  |  |  |
| **6** | **h** | Normal Chow: 10 | independently treated animals | one aortic root section and image per biological replicate | **Kruskal-Wallis test with Dunn's multiple comparisons test** | Normal Chow vs. Pcsk9-AAV8/HFD + DMSO: 0.0008 |
|  |  | Pcsk9-AAV8/HFD + DMSO: 12 |  |  |  | Pcsk9-AAV8/HFD + DMSO vs. Pcsk9-AAV8/HFD + KL-2: 0.0237 |
|  |  | Pcsk9-AAV8/HFD + KL-2: 8 |  |  |  |  |
| **6** | **j** | Normal Chow: 5 | independently treated animals | one aortic root section and image per biological replicate | **Kruskal-Wallis test with Dunn's multiple comparisons test** | Normal Chow vs. Pcsk9-AAV8/HFD + DMSO: 0.0001 |
|  |  | Pcsk9-AAV8/HFD + DMSO: 11 |  |  |  | Pcsk9-AAV8/HFD + DMSO vs. Pcsk9-AAV8/HFD + KL-2: 0.0337 |
|  |  | Pcsk9-AAV8/HFD + KL-2: 7 |  |  |  |  |
| **6** | **m** | Normal Chow: 6 | independently treated animals | one serum per biological replicate | **Kruskal-Wallis test with Dunn's multiple comparisons test** | Normal Chow vs. Pcsk9-AAV8/HFD + DMSO: < 0.0001 |
|  |  | Pcsk9-AAV8/HFD + DMSO: 6 |  |  |  | Normal Chow vs. Pcsk9-AAV8/HFD + KL-2: < 0.0001 |
|  |  | Pcsk9-AAV8/HFD + KL-2: 6 |  |  |  |  |
| **6** | **o** | Normal Chow: 7 | independently treated animals | one aorta and image per biological replicate | **Kruskal-Wallis test with Dunn's multiple comparisons test** | Normal Chow vs. Pcsk9-AAV8/HFD + DMSO: 0.0002 |
|  |  | Pcsk9-AAV8/HFD + DMSO: 6 |  |  |  | Pcsk9-AAV8/HFD + DMSO vs. Pcsk9-AAV8/HFD + KL-2: 0.0006 |
|  |  | Pcsk9-AAV8/HFD + KL-2: 6 |  |  |  |  |
| **6** | **q** | Normal Chow: 6 | independently treated animals | one aortic root section and image per biological replicate | **One-way ANOVA with Dunnett's multiple comparisons test, with a single pooled variance** | Normal Chow vs. Pcsk9-AAV8/HFD + DMSO: 0.0002 |
|  |  | Pcsk9-AAV8/HFD + DMSO: 7 |  |  |  | Pcsk9-AAV8/HFD + DMSO vs. Pcsk9-AAV8/HFD + KL-2: 0.00374 |
|  |  | Pcsk9-AAV8/HFD + KL-2: 7 |  |  |  |  |
| **6** | **r** | Normal Chow: 6 | independently treated animals | one aortic root section and image per biological replicate | **Kruskal-Wallis test with Dunn's multiple comparisons test** | Normal Chow vs. Pcsk9-AAV8/HFD + DMSO: < 0.0001 |
|  |  | Pcsk9-AAV8/HFD + DMSO: 7 |  |  |  | Pcsk9-AAV8/HFD + DMSO vs. Pcsk9-AAV8/HFD + KL-2: < 0.0001 |
|  |  | Pcsk9-AAV8/HFD + KL-2: 7 |  |  |  |  |
| **6** | **t** | Normal Chow: 6 | independently treated animals | one aortic root section and image per biological replicate | **Kruskal-Wallis test with Dunn's multiple comparisons test** | Normal Chow vs. Pcsk9-AAV8/HFD + DMSO: < 0.0001 |
|  |  | Pcsk9-AAV8/HFD + DMSO: 7 |  |  |  | Pcsk9-AAV8/HFD + DMSO vs. Pcsk9-AAV8/HFD + KL-2: 0.0002 |
|  |  | Pcsk9-AAV8/HFD + KL-2: 7 |  |  |  |  |
|  |  |  |  |  |  |  |
| **Figure** | **Panel** | **Biological n** | **Independent replicate definition** | **Quantification unit** | **statistical test used** | **exact p-value** |
| **7** | **b** | Normal Chow: 5 | independently treated animals | one aortic root section and image per biological replicate | **Kruskal-Wallis test with Dunn's multiple comparisons test** | Normal Chow vs. Pcsk9-AAV8/HFD + DMSO: 0.0015 |
|  |  | Pcsk9-AAV8/HFD + DMSO: 10 |  |  |  | Pcsk9-AAV8/HFD + DMSO vs. Pcsk9-AAV8/HFD + KL-2: 0.0258 |
|  |  | Pcsk9-AAV8/HFD + KL-2: 5 |  |  |  |  |
| **7** | **d** | Normal Chow: 5 | independently treated animals | one aortic root section and image per biological replicate | **Kruskal-Wallis test with Dunn's multiple comparisons test** | Normal Chow vs. Pcsk9-AAV8/HFD + DMSO: 0.0233 |
|  |  | Pcsk9-AAV8/HFD + DMSO: 11 |  |  |  | Pcsk9-AAV8/HFD + DMSO vs. Pcsk9-AAV8/HFD + KL-2: 0.0194 |
|  |  | Pcsk9-AAV8/HFD + KL-2: 7 |  |  |  |  |
| **7** | **e** | Normal Chow: 6 | independently treated animals | one cDNA of one apex cordis per biological replicate | **t-test** | Normal Chow vs. Pcsk9-AAV8/HFD + DMSO: 0.0455 |
|  |  | Pcsk9-AAV8/HFD + DMSO: 6 |  |  |  | Pcsk9-AAV8/HFD + DMSO vs. Pcsk9-AAV8/HFD + KL-2: 0.0345 |
|  |  | Pcsk9-AAV8/HFD + KL-2: 8 |  |  |  |  |
| **7** | **f** | Normal Chow: 6 | independently treated animals | one plasma per biological replicate | **One-way ANOVA with Dunnett's multiple comparisons test, with a single pooled variance** | Normal Chow vs. Pcsk9-AAV8/HFD + DMSO: 0.0006 |
|  |  | Pcsk9-AAV8/HFD + DMSO: 6 |  |  |  | Pcsk9-AAV8/HFD + DMSO vs. Pcsk9-AAV8/HFD + KL-2: 0.0017 |
|  |  | Pcsk9-AAV8/HFD + KL-2: 8 |  |  |  |  |
| **7** | **g** | less vulnerable atherosclerotic plaques: 703 | independent human patients | one plaque from one human patient per biological replicate | **generalized linear model; PVI evaluated using Spearmans rank correlation coefficient** | p<0.05 |
|  |  | more vulnerable atherosclerotic plaques: 345 |  |  |  |  |
| **7** | **h** | less vulnerable atherosclerotic plaques: 703 | independent human patients | one plaque from one human patient per biological replicate | **generalized linear model** | p<0.05 |
|  |  | more vulnerable atherosclerotic plaques: 345 |  |  |  |  |
| **7** | **j** | Normal Chow: 6 | independently treated animals | one aortic root section and image per biological replicate | **One-way ANOVA with Dunnett's multiple comparisons test, with a single pooled variance** | Normal Chow vs. Pcsk9-AAV8/HFD + DMSO: < 0.0001 |
|  |  | Pcsk9-AAV8/HFD + DMSO: 7 |  |  |  | Pcsk9-AAV8/HFD + DMSO vs. Pcsk9-AAV8/HFD + KL-2: 0.0276 |
|  |  | Pcsk9-AAV8/HFD + KL-2: 7 |  |  |  |  |
| **7** | **l** | Normal Chow: 6 | independently treated animals | one aortic root section and image per biological replicate | **One-way ANOVA with Dunnett's multiple comparisons test, with a single pooled variance** | Normal Chow vs. Pcsk9-AAV8/HFD + DMSO: < 0.0001 |
|  |  | Pcsk9-AAV8/HFD + DMSO: 7 |  |  |  | Pcsk9-AAV8/HFD + DMSO vs. Pcsk9-AAV8/HFD + KL-2: 0.0008 |
|  |  | Pcsk9-AAV8/HFD + KL-2: 7 |  |  |  |  |
|  |  |  |  |  |  |  |
|  |  |  |  |  |  |  |
| **Supplementary Figures** | | |  |  |  |  |
|  |  |  |  |  |  |  |
| **Figure** | **Panel** | **Biological n** | **Independent replicate definition** | **Quantification unit** | **statistical test used** | **exact p-value** |
| **S1** | **d** | Control: 4 | independently treated cell cultures | one bulk RNA-seq sample per biological replicate | **Negative binomial exact test** | p<0.05 |
|  |  | EndMT, 1-Hit: 4 |  |  |  |  |
|  |  | EndMT, 2-Hit: 4 |  |  |  |  |
| **S1** | **e** | Control: 4 | independently treated cell cultures | one cDNA sample per biological replicate | **t-test** | Control vs. EndMT: < 0.0001 |
|  |  | EndMT: 4 |  |  |  |  |
| **S1** | **f** | Control: 4 | independently treated cell cultures | one cDNA sample per biological replicate | **t-test** | Control vs. EndMT: < 0.0001 |
|  |  | EndMT: 4 |  |  |  |  |
| **S1** | **g** | Control: 4 | independently treated cell cultures | one cDNA sample per biological replicate | **t-test** | Control vs. EndMT: 0.0087 |
|  |  | EndMT: 4 |  |  |  |  |
| **S1** | **h** | Control: 4 | independently treated cell cultures | one cDNA sample per biological replicate | **Mann-Whitney test** | Control vs. EndMT: 0.0143 |
|  |  | EndMT: 4 |  |  |  |  |
| **S1** | **i** | Control: 3 | independently treated cell cultures | one cDNA sample per biological replicate | **t-test** | Control vs. EndMT: 0.0208 |
|  |  | EndMT: 3 |  |  |  |  |
| **S1** | **j** | Control: 3 | independently treated cell cultures | one cDNA sample per biological replicate | **t-test** | Control vs. EndMT: 0.0377 |
|  |  | EndMT: 3 |  |  |  |  |
| **S1** | **l** | Control: 5 | independently treated cell cultures | one immunoblot sample and image per biological replicate | **Mann-Whitney test** | Control vs. EndMT: 0.0159 |
|  |  | EndMT: 5 |  |  |  |  |
| **S1** | **m** | Control: 5 | independently treated cell cultures | one immunoblot sample and image per biological replicate | **t-test** | Control vs. EndMT: 0.0122 |
|  |  | EndMT: 5 |  |  |  |  |
|  |  |  |  |  |  |  |
| **Figure** | **Panel** | **Biological n** | **Independent replicate definition** | **Quantification unit** | **statistical test used** | **exact p-value** |
| **S4** | **a** | Normal Chow: 5 | independently treated animals | one bulk RNA-seq sample per biological replicate | **One-way ANOVA with Dunnett's multiple comparisons test, with a single pooled variance** | Normal Chow vs. Pcsk9-AAV8/HFD + DMSO: 0.5387 |
|  |  | Pcsk9-AAV8/HFD + DMSO: 7 |  |  |  | Pcsk9-AAV8/HFD + DMSO vs. Pcsk9-AAV8/HFD + KL-2: 0.9551 |
|  |  | Pcsk9-AAV8/HFD + KL-2: 8 |  |  |  |  |
| **S4** | **b** | Normal Chow: 5 | independently treated animals | one bulk RNA-seq sample per biological replicate | **One-way ANOVA with Dunnett's multiple comparisons test, with a single pooled variance** | Normal Chow vs. Pcsk9-AAV8/HFD + DMSO: 0.7245 |
|  |  | Pcsk9-AAV8/HFD + DMSO: 7 |  |  |  | Pcsk9-AAV8/HFD + DMSO vs. Pcsk9-AAV8/HFD + KL-2: 0.9519 |
|  |  | Pcsk9-AAV8/HFD + KL-2: 8 |  |  |  |  |
| **S4** | **c** | Normal Chow: 5 | independently treated animals | one bulk RNA-seq sample per biological replicate | **Kruskal-Wallis test with Dunn's multiple comparisons test** | Normal Chow vs. Pcsk9-AAV8/HFD + DMSO: 0.1607 |
|  |  | Pcsk9-AAV8/HFD + DMSO: 7 |  |  |  | Pcsk9-AAV8/HFD + DMSO vs. Pcsk9-AAV8/HFD + KL-2: 0.2648 |
|  |  | Pcsk9-AAV8/HFD + KL-2: 8 |  |  |  |  |
| **S4** | **h** | Normal Chow: 4 | independently treated animals | one bulk RNA-seq sample per biological replicate | **One-way ANOVA with Dunnett's multiple comparisons test, with a single pooled variance** | Normal Chow vs. Pcsk9-AAV8/HFD + DMSO: 0.0261 |
|  |  | Pcsk9-AAV8/HFD + DMSO: 4 |  |  |  |  |
|  |  | Pcsk9-AAV8/HFD + KL-2: 3 |  |  |  |  |
| **S4** | **i** | Normal Chow: 4 | independently treated animals | one bulk RNA-seq sample per biological replicate | **One-way ANOVA with Dunnett's multiple comparisons test, with a single pooled variance** | Normal Chow vs. Pcsk9-AAV8/HFD + DMSO: 0.0131 |
|  |  | Pcsk9-AAV8/HFD + DMSO: 4 |  |  |  |  |
|  |  | Pcsk9-AAV8/HFD + KL-2: 3 |  |  |  |  |
| **S4** | **j** | Normal Chow: 4 | independently treated animals | one bulk RNA-seq sample per biological replicate | **One-way ANOVA with Dunnett's multiple comparisons test, with a single pooled variance** | Pcsk9-AAV8/HFD + DMSO vs. Pcsk9-AAV8/HFD + KL-2: < 0.0001 |
|  |  | Pcsk9-AAV8/HFD + DMSO: 4 |  |  |  |  |
|  |  | Pcsk9-AAV8/HFD + KL-2: 3 |  |  |  |  |
| **S4** | **k** | Normal Chow: 4 | independently treated animals | one bulk RNA-seq sample per biological replicate | **One-way ANOVA with Dunnett's multiple comparisons test, with a single pooled variance** | Pcsk9-AAV8/HFD + DMSO vs. Pcsk9-AAV8/HFD + KL-2: 0.0080 |
|  |  | Pcsk9-AAV8/HFD + DMSO: 4 |  |  |  |  |
|  |  | Pcsk9-AAV8/HFD + KL-2: 3 |  |  |  |  |
| **S4** | **l** | Normal Chow: 4 | independently treated animals | one bulk RNA-seq sample per biological replicate | **One-way ANOVA with Dunnett's multiple comparisons test, with a single pooled variance** | Pcsk9-AAV8/HFD + DMSO vs. Pcsk9-AAV8/HFD + KL-2: 0.0069 |
|  |  | Pcsk9-AAV8/HFD + DMSO: 4 |  |  |  |  |
|  |  | Pcsk9-AAV8/HFD + KL-2: 3 |  |  |  |  |
| **S4** | **m** | Normal Chow: 4 | independently treated animals | one bulk RNA-seq sample per biological replicate | **One-way ANOVA with Dunnett's multiple comparisons test, with a single pooled variance** | Pcsk9-AAV8/HFD + DMSO vs. Pcsk9-AAV8/HFD + KL-2: 0.0002 |
|  |  | Pcsk9-AAV8/HFD + DMSO: 4 |  |  |  |  |
|  |  | Pcsk9-AAV8/HFD + KL-2: 3 |  |  |  |  |
|  |  |  |  |  |  |  |
| **Figure** | **Panel** | **Biological n** | **Independent replicate definition** | **Quantification unit** | **statistical test used** | **exact p-value** |
| **S5** | **b** | Normal Chow: | independently treated animals | one aortic root section and image per biological replicate | **t-test** | Pcsk9-AAV8/HFD + DMSO vs. Pcsk9-AAV8/HFD + KL-2: 0.0393 |
|  |  | Pcsk9-AAV8/HFD + DMSO: 5 |  |  |  |  |
|  |  | Pcsk9-AAV8/HFD + KL-2: 7 |  |  |  |  |
|  |  |  |  |  |  |  |
| **Figure** | **Panel** | **Biological n** | **Independent replicate definition** | **Quantification unit** | **statistical test used** | **exact p-value** |
| **S6** | **b** | Normal Chow: 5 | independently treated animals | one aortic root section and image per biological replicate | **One-way ANOVA with Dunnett's multiple comparisons test, with a single pooled variance** | Normal Chow vs. Pcsk9-AAV8/HFD + DMSO: 0.0004 |
|  |  | Pcsk9-AAV8/HFD + DMSO: 10 |  |  |  | Pcsk9-AAV8/HFD + DMSO vs. Pcsk9-AAV8/HFD + KL-2: 0.0183 |
|  |  | Pcsk9-AAV8/HFD + KL-2: 8 |  |  |  |  |
| **S6** | **d** | Normal Chow: 5 | independently treated animals | one aortic root section and image per biological replicate | **Kruskal-Wallis test with Dunn's multiple comparisons test** | Normal Chow vs. Pcsk9-AAV8/HFD + DMSO: 0.0018 |
|  |  | Pcsk9-AAV8/HFD + DMSO: 10 |  |  |  | Pcsk9-AAV8/HFD + DMSO vs. Pcsk9-AAV8/HFD + KL-2: 0.0374 |
|  |  | Pcsk9-AAV8/HFD + KL-2: 7 |  |  |  |  |
| **S6** | **f** | Normal Chow: 5 | independently treated animals | one aortic root section and image per biological replicate | **One-way ANOVA with Dunnett's multiple comparisons test, with a single pooled variance** | Normal Chow vs. Pcsk9-AAV8/HFD + DMSO: 0.0015 |
|  |  | Pcsk9-AAV8/HFD + DMSO: 10 |  |  |  | Pcsk9-AAV8/HFD + DMSO vs. Pcsk9-AAV8/HFD + KL-2: 0.0318 |
|  |  | Pcsk9-AAV8/HFD + KL-2: 8 |  |  |  |  |
